# Inhibiting NLRP3 inflammasome activation prevents copper-induced neuropathology in a murine model of Wilson’s disease

**DOI:** 10.1038/s41419-021-03397-1

**Published:** 2021-01-18

**Authors:** Jianjian Dong, Xun Wang, Chenchen Xu, Manli Gao, Shijing Wang, Jin Zhang, Haiyang Tong, Lulu Wang, Yongzhu Han, Nan Cheng, Yongsheng Han

**Affiliations:** 1 High Magnetic Field Laboratory, Hefei Institutes of Physical Science, Chinese Academy of Sciences, Hefei, 230031 P. R. China; 2 University of Science and Technology of China, Hefei, 230026 P. R. China; 3 The Affiliated Hospital of the Neurology Institute, Anhui University of Chinese Medicine, Hefei, 230061 P. R. China; 4 Anhui University of Chinese Medicine, Hefei, 230012 P. R. China

**Keywords:** Inflammasome, Neuroimmunology

## Abstract

Wilson’s disease (WD) is an inherited disorder characterized by excessive accumulation of copper in the body, particularly in the liver and brain. In the central nervous system (CNS), extracellular copper accumulation triggers pathological microglial activation and subsequent neurotoxicity. Growing evidence suggests that levels of inflammatory cytokines are elevated in the brain of murine WD models. However, the mechanisms associated with copper deposition to neuroinflammation have not been completely elucidated. In this study, we investigated how the activation of NLR family pyrin domain containing 3 (NLRP3) inflammasome contributes to copper-mediated neuroinflammation in an animal model of WD. Elevated levels of interleukin-1β, interleukin-18, interleukin-6, and tumor necrosis factor-α were observed in the sera of WD patients and toxic milk (TX) mice. The protein levels of inflammasome adaptor molecule apoptosis-associated speck-like protein containing a C-terminal caspase recruitment domain (ASC), cleaved caspase-1, and interleukin-1β were upregulated in the brain regions of the TX mice. The NLRP3 inflammasome was activated in the TX mice brains. Furthermore, the activation of NLRP3 inflammasome was noted in primary microglia treated with CuCl_2_, accompanied by the increased levels of cleaved caspase-1, ASC, and interleukin-1β. Blocking NLRP3 inflammasome activation with siNlrp3 or MCC950 reduced interleukin-1β and interleukin-18 production, thereby effectively mitigating cognitive decline, locomotor behavior impairment, and neurodegeneration in TX mice. Overall, our study demonstrates the contribution of copper overload-mediated activation of NLRP3 inflammasome to progressive neuropathology in the CNS of a murine model of WD. Therefore, blockade of the NLRP3 inflammasome activation could be a potential therapeutic strategy for WD.

## Introduction

Wilson’s disease (WD) is an autosomal recessive disorder related to copper metabolism that is caused by mutations in *ATP7B*, which mediates the binding of copper to ceruloplasmin and the excretion of copper into bile^1^. *ATP7B* dysfunction results in excess copper deposits in various organs, including the liver, brain, and eyes. Growing evidence indicates that neuroinflammation is common in patients with WD^2–4^. The pathological changes in the central nervous system (CNS) associated with WD include astrogliosis, demyelination, and tissue disintegration^5,6^. However, the mechanism by which copper overload contributes to progressive neurodegeneration and neuroinflammation in WD remains elusive.

Inflammasomes are large multiprotein complexes that act as intracellular sensors for a variety of danger-associated molecular patterns and pathogen-associated molecular patterns (PAMPs)^7,8^. Canonical inflammasomes are composed of an inflammasome sensor protein bound to pro-caspase-1, usually via an adaptor molecule apoptosis-associated speck-like protein containing a C-terminal caspase recruitment domain (ASC). Inflammasome sensor proteins include nucleotide binding oligomerization domain (NOD)-like receptor family, pyrin domain-containing protein (NLRP) 1, NLRP3, NLRP6, neuronal apoptosis inhibitory protein (NAIP)/ NLR containing C-terminal caspase recruitment domain protein 4 (NLRC4), absent in melanoma 2 (AIM2), and pyrin; each of these responds to specific pathogen- and danger-associated signals indicative of infection or cellular damage, triggering sensor protein oligomerization and the recruitment of ASC^9,10^. Activation of the canonical inflammasomes promotes caspase-1-dependent maturation of interleukin (IL)-1β and IL-18^11,12^.

IL-1β, a proinflammatory cytokine that is upregulated in many CNS inflammatory conditions, including Alzheimer’s disease, Parkinson’s disease, multiple sclerosis, and other neurodegenerative disorders, is a master regulator of immune responses in the CNS^13,14^. It is capable of activating an innate immune response by inducing the expression of various inflammatory cytokines and chemokines and eliciting leukocyte recruitment^15,16^. As a key driver of the neuroinflammatory process, IL-1β is expressed by many different cell types, including microglia, astrocytes, endothelial cells, recruited leukocytes, and oligodendrocytes, within the CNS. The IL-1β precursor (namely, pro-IL-1β) is not constitutively expressed and is induced by NF-κB-mediated transcription activation. In addition, pro-IL-1β, is not biologically active; however, it requires proteolytic processing by caspase-1, resulting in the secretion of mature IL-1β^17,18^. Caspase-1, the predominant IL-1 processing protease, is abundantly present in the microglia as a proenzyme and is typically activated by inflammasomes^19–21^.

Although copper plays an essential role in the development of healthy nerves and other physiological functions in the CNS^22^, the accumulation of excessive Cu^2+^ amounts can lead to neuroinflammatory injury^23,24^. WD results from copper accumulation in the liver and brain caused by mutations in *ATP7B*, encoding a copper transporter. Copper accumulation in rodent brains induces WD features and the recruitment of inflammatory cells into the affected brain region^4^. Copper is specifically required for inflammasome activation in macrophages, as the depletion of bioavailable copper results in an attenuated caspase-1-dependent inflammation and reduced susceptibility to lipopolysaccharide (LPS)-induced septic shock^25^. Therefore, we hypothesized that copper accumulation activates NLRP3 inflammasome to mediate neuroinflammation in the brain.

The present study aimed to investigate the role of inflammasome activation in the initiation of WD using toxic milk (TX) mice, an animal model for WD. Herein, we show that the activation of NLRP3 inflammasome in the brain of TX mice caused copper-induced neuropathology. siRNA silencing or pharmacologic blockade of NLRP3 inflammasome activation inhibited the microglial activation and release of proinflammatory cytokines in the CNS, thereby improving cognitive function in TX mice. Altogether, our study highlighted the importance of NLRP3 activation as a pathological mechanism involved in the development of WD and suggested that inhibiting of the activation of NLRP3 inflammasome might be an ideal therapeutic strategy to treat CNS complications in WD.

## Results

### Activation of the NLRP3 inflammasome is associated with WD

**Fig. 1:**
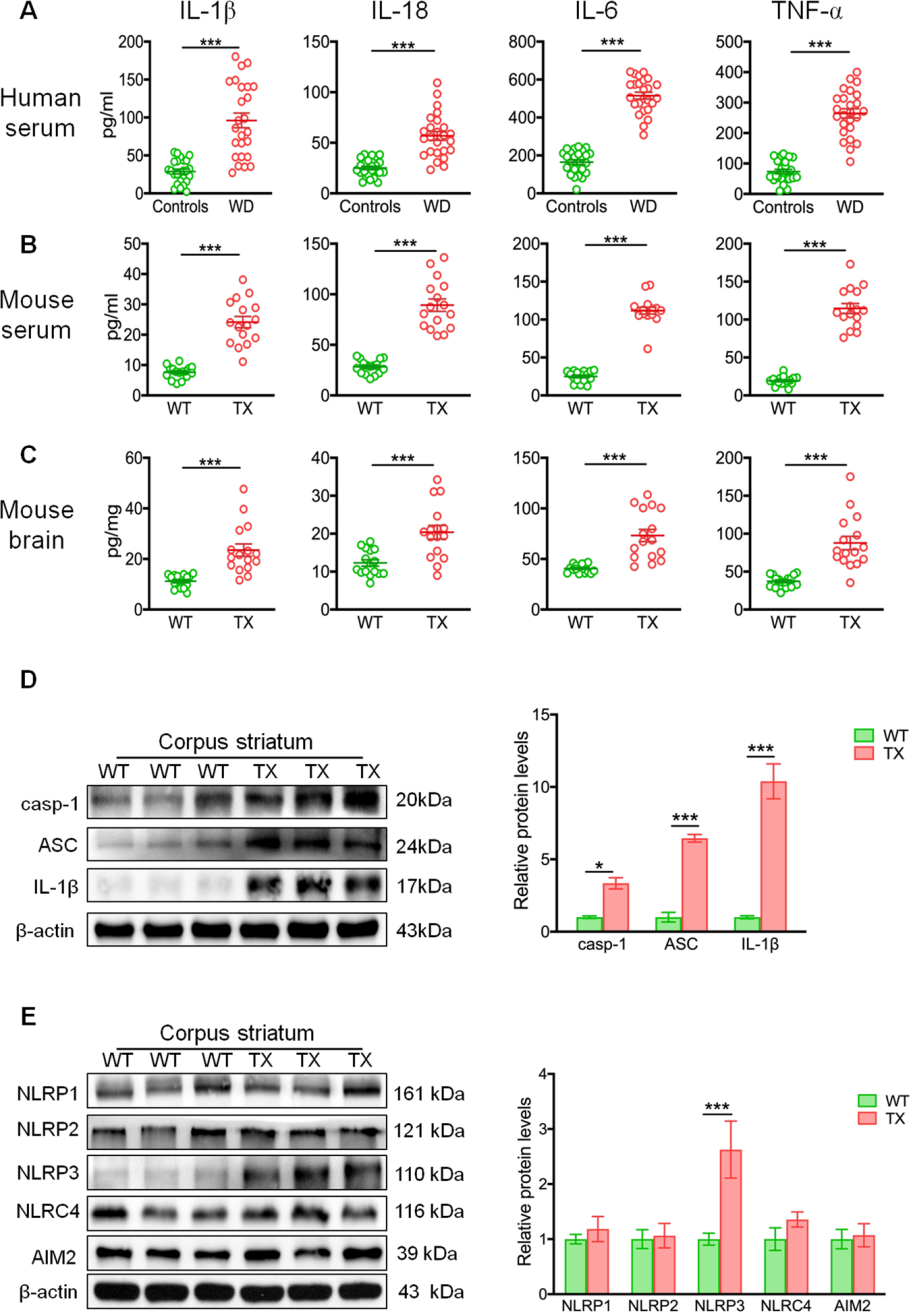
Activation of NLRP3 inflammasome in patients with Wilson’s disease (WD) and the WD animal model. **a** Levels of circulating interleukin (IL)-1β, IL-18, IL-6, and tumor necrosis factor (TNF)-α in the serum of healthy controls and patients with WD (*n* = 25). **b** Serum IL-1β, IL-18, IL-6, and TNF-α levels in toxic milk (TX) mice and wild type (WT) mice (pg/ml) (*n* = 16). **c** IL-1β, IL-18, IL-6, and TNF-α levels in the brain of TX mice and WT controls mice (pg/mg) (*n* = 16). **d** Levels of cleaved caspase-1 (casp-1), ASC, and IL-1β in the corpus striatum of TX mice and WT mice were quantified using western blotting (*n* = 6). **e** Levels of NLRP1, NLRP2, NLRP3, NLRC4, and AIM2 levels in the corpus striatum of TX mice and WT controls were measured using western blotting (*n* = 6). Data are presented as mean ± SEM; Student’s *t* test, ^*^P < 0.05, ^***^P < 0.001 versus the corresponding control or WT mice.

To determine if inflammation and inflammasome activation are involved in the development of WD, we first compared the levels of inflammatory cytokines in the sera of WD patients with those in healthy controls. Our data revealed significantly higher levels of circulating IL-1β, IL-18, IL-6, and TNF-α in the sera of WD patients (Fig. 1a). In addition, elevated levels of IL-1β, IL-18, IL-6, and TNF-α were also noted in the sera (Fig. 1b) and brain (Fig. 1c) of TX mice, as compared to those in WT mice. Furthermore, the levels of caspase-1, ASC, and IL-1β were remarkably elevated in the corpus striatum (Fig. 1d), hippocampus (Fig. S1a), cortex (Fig. S1b), and cerebellum (Fig. S1c) of the TX mice. Notably, the levels of NLRP3 were also remarkably higher in the corpus striatum (Fig. 1e) and hippocampus (Fig. S2a), while levels of NLRP2 and NLRP3 were elevated in the cortex (Fig. S2b) and cerebellum (Fig. S2c) of TX mice, compared to the WT mice. Mechanistically, the expression of *Nlrp3, Casp1*, and *ASC* was significantly elevated in the brains of TX mice, as compared with those in WT mice (Fig. S3). These findings demonstrated that inflammasome components are upregulated and subsequently activated in the early stages of neurodegeneration in TX mice.

### Copper induces NLRP3 activation and extracellular ASC release in microglia

**Fig. 2:**
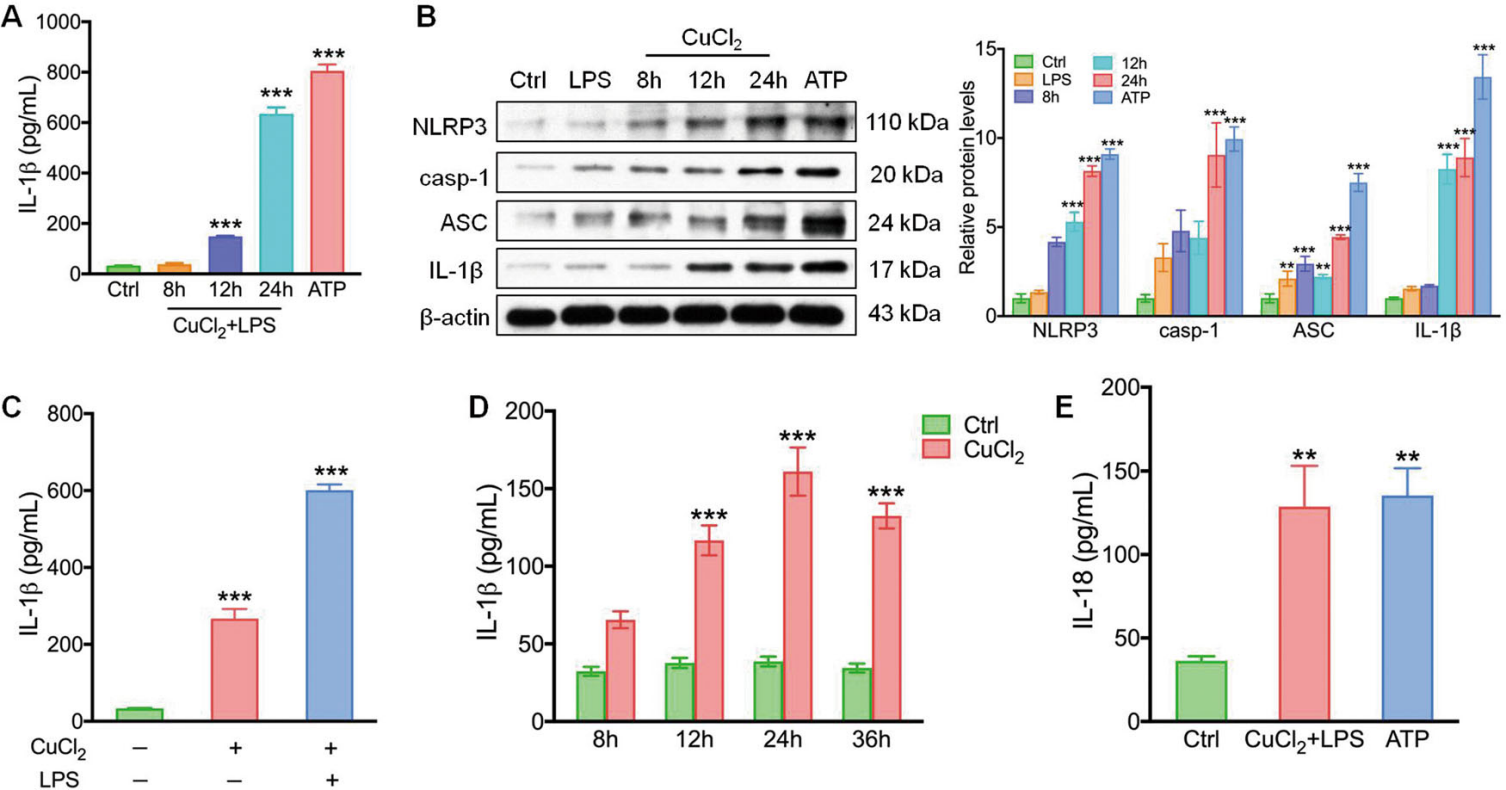
CuCl_2_ induces interleukin (IL-1β) and IL-18 production and NLRP3 inflammasome activation. **a** Primary microglia were treated with CuCl_2_ (10 µM) and lipopolysaccharide (LPS, 200 ng/ml), supernatants were collected at 8, 12, and 24 h, and levels of IL-1β were quantified with ELISA; ATP (5 mM) treatment served as the positive control. **b** Primary microglia were treated with CuCl_2_ for 8, 12, and 24 h, and the protein levels of NLRP3, cleaved caspase-1 (casp-1), ASC, and IL-1β were quantified using western blotting; ATP (5 mM) treatment served as positive control. **c** Primary microglia were treated with or without CuCl_2_ (10 µM) and LPS (200 ng/ml) for 24 h, and levels of IL-1β were subsequently quantified using ELISA. **d** Primary microglia were treated with or without CuCl_2_ (10 µM) for 8, 12, 24, and 36 h, and the levels of IL-1β were quantified using ELISA. **e** The levels of IL-18 in microglia treated with CuCl_2_ and LPS for 24 h. Data are presented as mean ± SEM; one-way ANOVA, two-way ANOVA, ^**^P < 0.01, ^***^P < 0.001 versus the corresponding control group.

The activation of microglia plays a critical role in the development of chronic neuroinflammation. We examined whether copper triggered NLRP3 inflammasome activation in microglia. When primary microglia were treated with CuCl_2_, levels of IL-1β were elevated in the supernatant starting 12 h after treatment (Fig. 2a). Western blotting revealed elevated levels of NLRP3, cleaved caspase-1, ASC, and IL-1β in CuCl_2_-treated microglia in a time-dependent manner (Fig. 2b). To determine whether LPS is essential for copper-induced inflammasome activation, we examined whether CuCl_2_ alone could trigger IL-1β production in the absence of LPS. Surprisingly, CuCl_2_ alone was able to trigger the secretion of mature IL-1β (Fig. 2c) from the microglia in a time-dependent manner (Fig. 2d). Furthermore, increased production of mature IL-18, an essential inflammatory mediator, was also observed in CuCl_2_-treated microglia (Fig. 2e).

### In vivo NLRP3 silencing suppresses the copper-induced inflammatory response

**Fig. 3:**
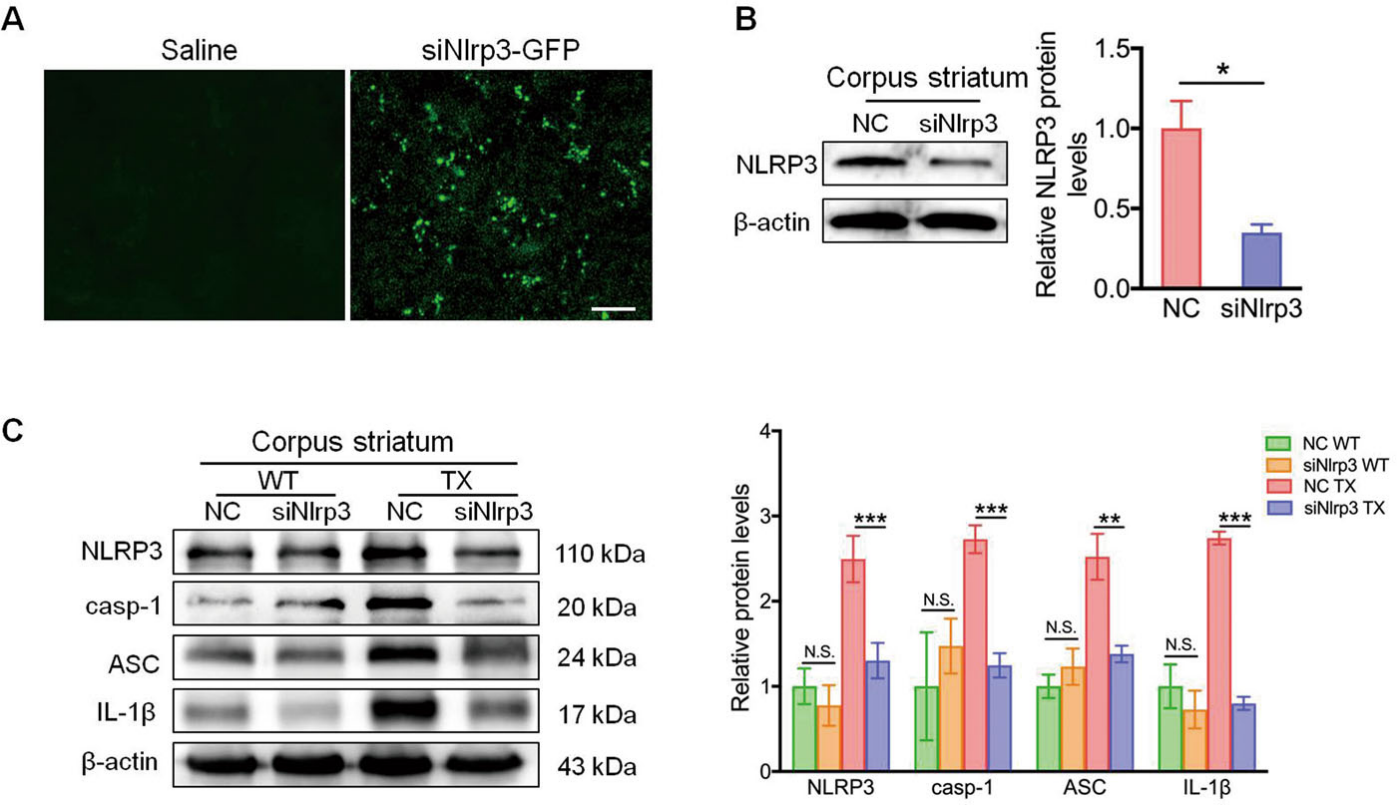
Silencing *Nlrp3* expression reduced the caspase-1 and interleukin (IL)-1β protein levels in the Wilson’s disease (WD) animal model. **a** Immunofluorescence staining of green fluorescent protein (GFP) in the corpus striatum of toxic milk (TX) mice injected with saline or Nlrp3 siRNA (siNlrp3). Scale bars, 50 µm. **b** Levels of NLRP3 in the brain of negative control siRNA (NC)- or siNlrp3-injected TX mice were measured using western blotting (*n* = 3). **c** Levels of NLRP3, cleaved caspase-1 (casp-1), ASC, and IL-1β in the corpus striatum of the NC- or siNlrp3-injected TX mice and WT mice were quantified using western blotting (*n* = 3). Data are presented as mean ± SEM; Student’s *t* test, two-way ANOVA, ^*^P < 0.05, ^**^P < 0.01, ^***^P < 0.001 versus the corresponding WT or NC group.

**Fig. 4:**
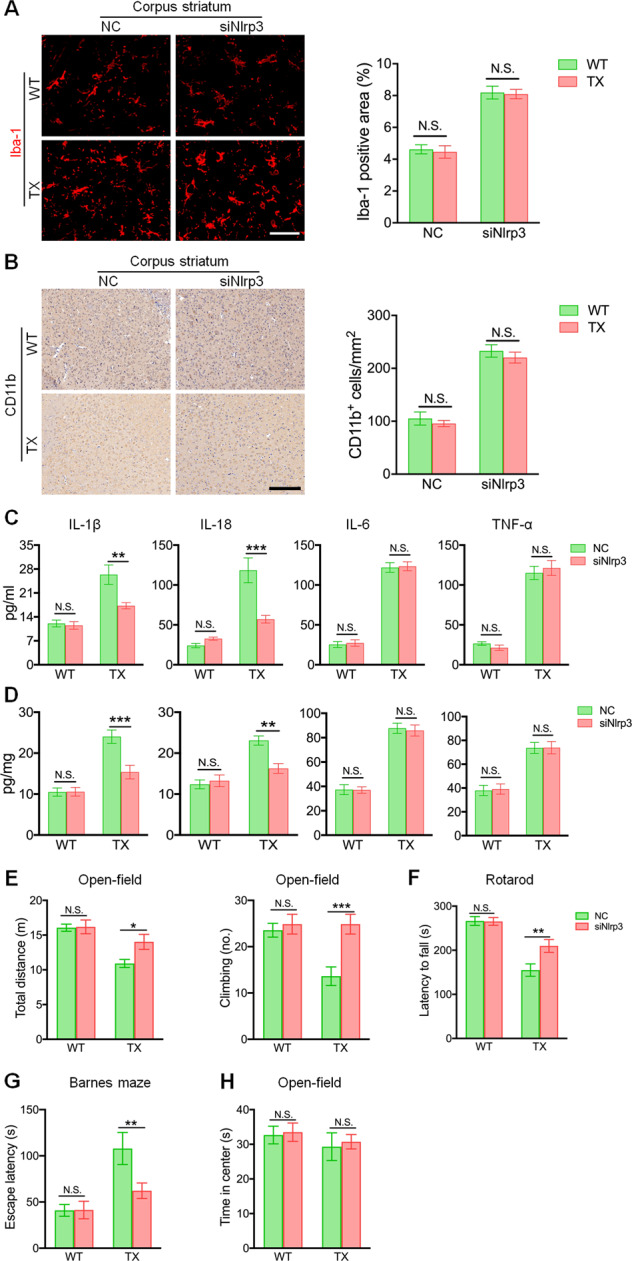
*Nlrp3* silencing inhibits interleukin (IL)-1β and IL-18 production, and reverses behavioral deficits in Wilson’s disease (WD) animal model. **a** Immunofluorescence staining and quantification of Iba-1-positive cells in the corpus striatum of the negative control siRNA (NC)- or the siNlrp3-injected toxic milk (TX) mice and wild type (WT) mice. Scale bars, 50 µm. **b** Immunohistochemical staining and quantification of CD11b-positive cells in the corpus striatum of the NC- or the siNlrp3-injected TX mice and WT mice. Scale bars, 200 µm. **c** Serum IL-1β, IL-18, IL-6, and tumor necrosis factor (TNF-α) levels in the NC- or the siNlrp3-injected TX mice and WT mice (pg/ml) (*n* = 6). **d** IL-1β, IL-18, IL-6, and TNF-α levels in the brain of the NC- or the siNlrp3-injected TX mice and WT mice (pg/mg) (*n* = 6). **e** TX mice or WT mice were treated with lentivirus-enveloped siNlrp3 or control virus; activities including total distance and climbing behaviors of open-field were measured (*n* = 15). **f** TX mice or WT mice were treated with lentivirus-enveloped siNlrp3 or control virus, and after 3 months, motor functions were tested using a rotarod test apparatus (*n* = 15). **g** Escape latency was measured using Barnes maze test (*n* = 15). **h** TX mice or WT mice were treated with lentivirus-enveloped siNlrp3 or control virus, and time spent in center of open-field was measured. (*n* = 15). Data are presented as mean ± SEM; two-way ANOVA, ^*^P < 0.05, ^**^P < 0.01, ^***^P < 0.001 versus the corresponding NC group.

To determine the importance of the NLRP3 inflammasome in copper-dependent inflammation in vivo, the expression of *Nlrp3* was interrupted by injecting lentivirus-enveloped siNlrp3 into TX mice through the tail vein (Fig. 3a). As expected, reduced levels of the NLRP3 protein were noted in the corpus striatum (Fig. 3b) of mice treated with lentivirus-enveloped siNlrp3, as well as in other areas of the brain (Fig. S4). Furthermore, reduced protein levels of cleaved caspase-1, ASC, and IL-1β were observed in the corpus striatum (Fig. 3c), hippocampus, cortex, and cerebellum (Fig. S4). Although the number of microglia in corpus striatum (Figs. 4a, b), hippocampus, cortex, and cerebellum (Fig. S5, S6) of siNlrp3-TX mice showed no significant differences with that of NC-treated TX mice, the levels of IL-1β and IL-18 (Fig. 4c, d) in TX-treated mice were significantly reduced following NLRP3 silencing. However, the levels of IL-6 and TNF-α showed no significant differences between siNlrp3-TX and NC-TX groups (Fig. 4c, d). Additionally, we examined whether siNlrp3 injection had beneficial effects on motor, emotional, and cognitive functions in TX mice. Interestingly, it increased the total distance and climbing behaviors (Fig. 4e), and improved motor function (Fig. 4f) and memory (Fig. 4g) of TX mice compared with NC-TX mice. However, siNlrp3 did not alter anxiety-like behavior in TX mice (Fig. 4h). Therefore, NLRP3 silencing suppresses copper-induced neuroinflammation and protects TX mice against progressive motor deficits and cognitive decline by eliminating NLRP3 inflammasome activation.

### MCC950 suppresses NLRP3 inflammasome activation in vitro

**Fig. 5:**
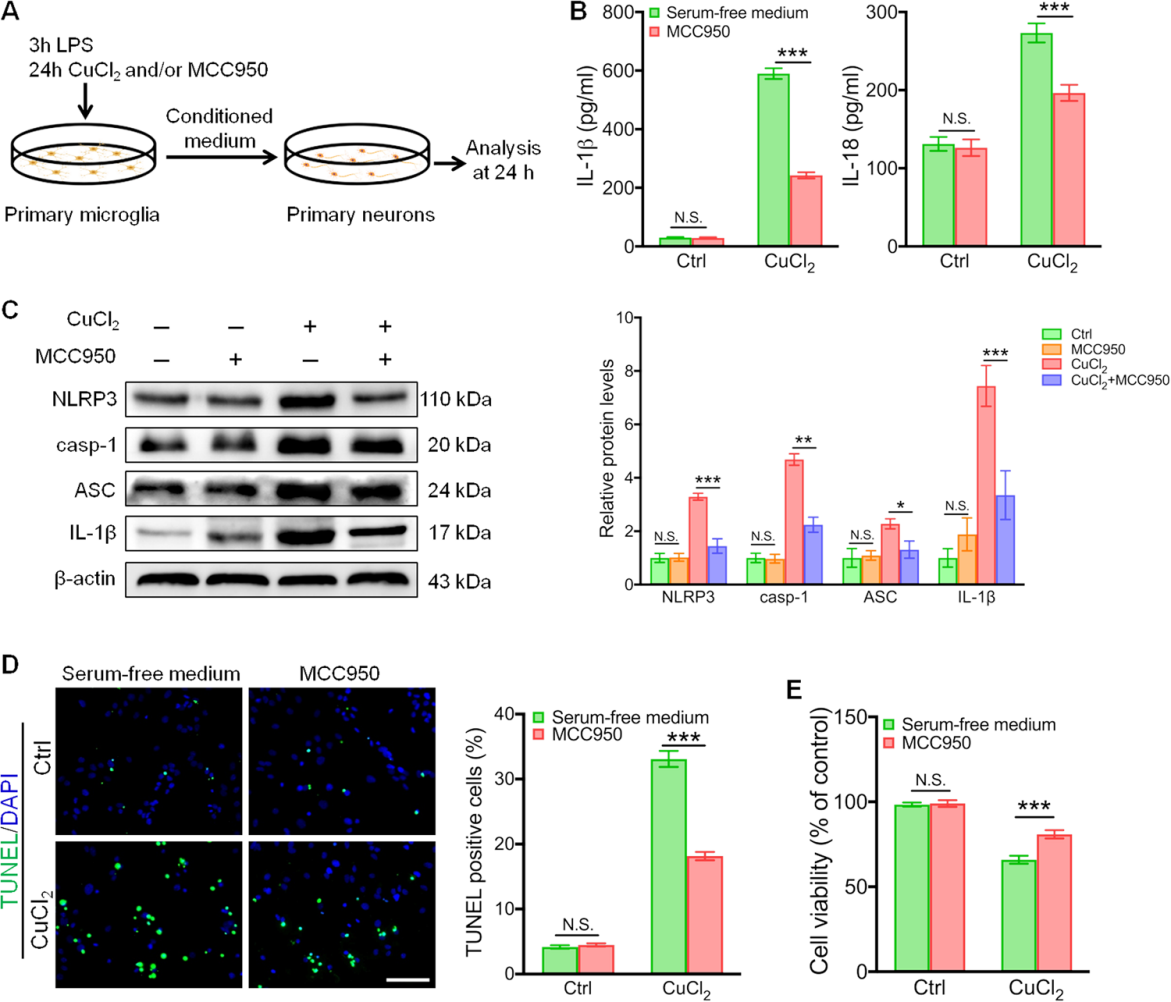
MCC950 inhibits NLRP3 inflammasome activation in CuCl_2_-induced microglia. **a** Schematic illustration of the experimental set-up. **b** Primary microglia were treated with or without CuCl_2_ (10 µM) and MCC950 (100 nM) for 24 h, and levels of interleukin (IL-1β) and IL-18 were quantified using ELISA (*n* = 6). **c** Primary microglia were treated with or without CuCl_2_ and MCC950, and the levels of NLRP3, cleaved caspase-1 (casp-1), ASC, and IL-1β were measured using western blotting (*n* = 4). **d** Primary neurons separated from the hippocampus were treated with conditioned medium from CuCl_2_- or MCC950-administrated microglia, and the apoptotic neuronal cells were quantified by TUNEL (green) immunofluorescence staining. Scale bars, 100 µm. **e** Cell viability of hippocampal neurons treated with conditioned medium from CuCl_2_- or MCC950-administrated microglia. Data are presented as mean ± SEM; two-way ANOVA, ^*^P < 0.05, ^**^P < 0.01, ^***^P < 0.001 versus the corresponding control group.

To examine whether pharmacologic blockade of NLRP3 inflammasome activation could inhibit microglial activation, we examined the inhibitory effect of MCC950, a potent inhibitor of NLRP3, on the activation of primary microglia induced by CuCl_2_ in vitro (Fig. 5a). CuCl_2_ stimuli increased the release of IL-1β and IL-18 in primary microglia. When added to the culture, MCC950 significantly reduced the levels of IL-1β and IL-18 secreted by CuCl_2_-activated microglia (Fig. 5b). The protein levels of NLRP3, caspase-1, ASC, and IL-1β in CuCl_2_-induced microglia were also attenuated by MCC950 treatment (Fig. 5c). Furthermore, conditioned medium was collected from primary microglia after LPS priming and CuCl_2_ or MCC950 treatment and subsequently added to primary neuron cultures. Compared to the control group, the TUNEL-positive cell number was significantly elevated in the culture treated with CuCl_2_ conditional medium. MCC950 conditional medium treatment decreased TUNEL-positive cell numbers in the neuron culture (Fig. 5d). In addition, MCC950 conditional medium efficiently reduced the number of dead neuron induced by CuCl_2_ (Fig. 5e). Together, MCC950 is sufficient to block the CuCl_2_-mediated pathological activation of microglial NLRP3 inflammasome.

### MCC950 protects against neurodegeneration in TX mice

**Fig. 6:**
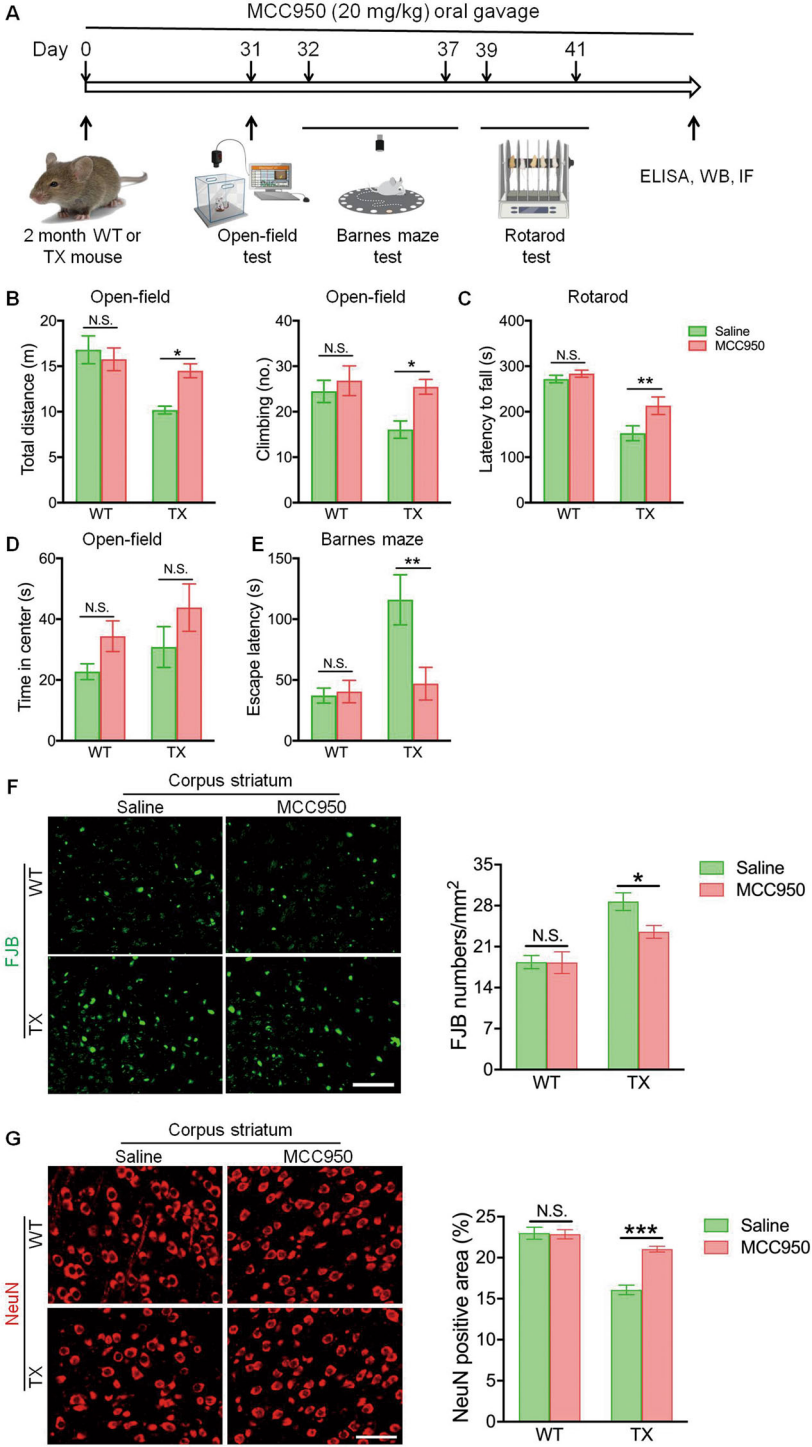
MCC950 treatment protects against behavior impairment and neurodegeneration in the Wilson’s disease (WD) animal model. **a** Schematic representation of MCC950 treatment experiments. **b**–**e** Toxic milk (TX) mice and wild type (WT) mice were treated with MCC950 or saline, (**b**) total distance and climbing behaviors were quantified with open-field tests (*n* = 15). **c** Motor functions were tested using a rotarod test apparatus (*n* = 15). **d** Time spent in center were quantified with open-field tests (*n* = 15). **e** Escape latency was measured using Barnes maze test (*n* = 15). **f** Fluorescence imaging of Fluoro-Jade B (FJB) staining (green) in the corpus striatum of TX mice and WT mice treated with MCC950 or saline. Scale bar, 50 µm. **g** Immunofluorescence staining and quantification of NeuN (red) in the corpus striatum of MCC950 or saline-treated TX mice and WT mice. Scale bar, 50 µm. Data are presented as mean ± SEM; two-way ANOVA, ^*^P < 0.05, ^**^P < 0.01, ^***^P < 0.001 versus the corresponding saline-treated group.

Previous studies have demonstrated that orally administered MCC950 crosses the blood–brain barrier and affects biological functions in the brain^26^. Therefore, the effects of MCC950 treatment on cognitive function, locomotor behavior, and anxiety in TX mice were examined (Fig. 6a). MCC950 treatment significantly enhanced the total distance spontaneously traveled by TX mice, compared saline-treated TX mice (Fig. 6b). Additionally, the decreased instances of climbing behaviors and motor function in TX mice were reversed by MCC950 (Figs. 6b, c). Time spent in the center zone did not differ among various groups (Fig. 6d). Barnes maze experiments revealed that MCC950-treated TX mice exhibited improved memory and spatial learning, compared with the saline-treated TX mice (Fig. 6e).

Furthermore, the neuroprotective effects of MCC950 were accessed by Fluoro-Jade B staining and NeuN staining. Copper deposition induced a neuronal loss in the corpus striatum (Fig. 6f, g), as well as in the hippocampus, cortex, and cerebellum of TX mice (Figs. S7, S8). However, neuron loss in TX mice was alleviated by MCC950 treatment (Figs. 6f, g). Therefore, MCC950 might protect against cognitive decline and locomotor behavior impairment by protecting neurodegeneration induced by copper deposition in TX mice.

### MCC950 reduces the activation of NLRP3 inflammasome in TX mice

**Fig. 7:**
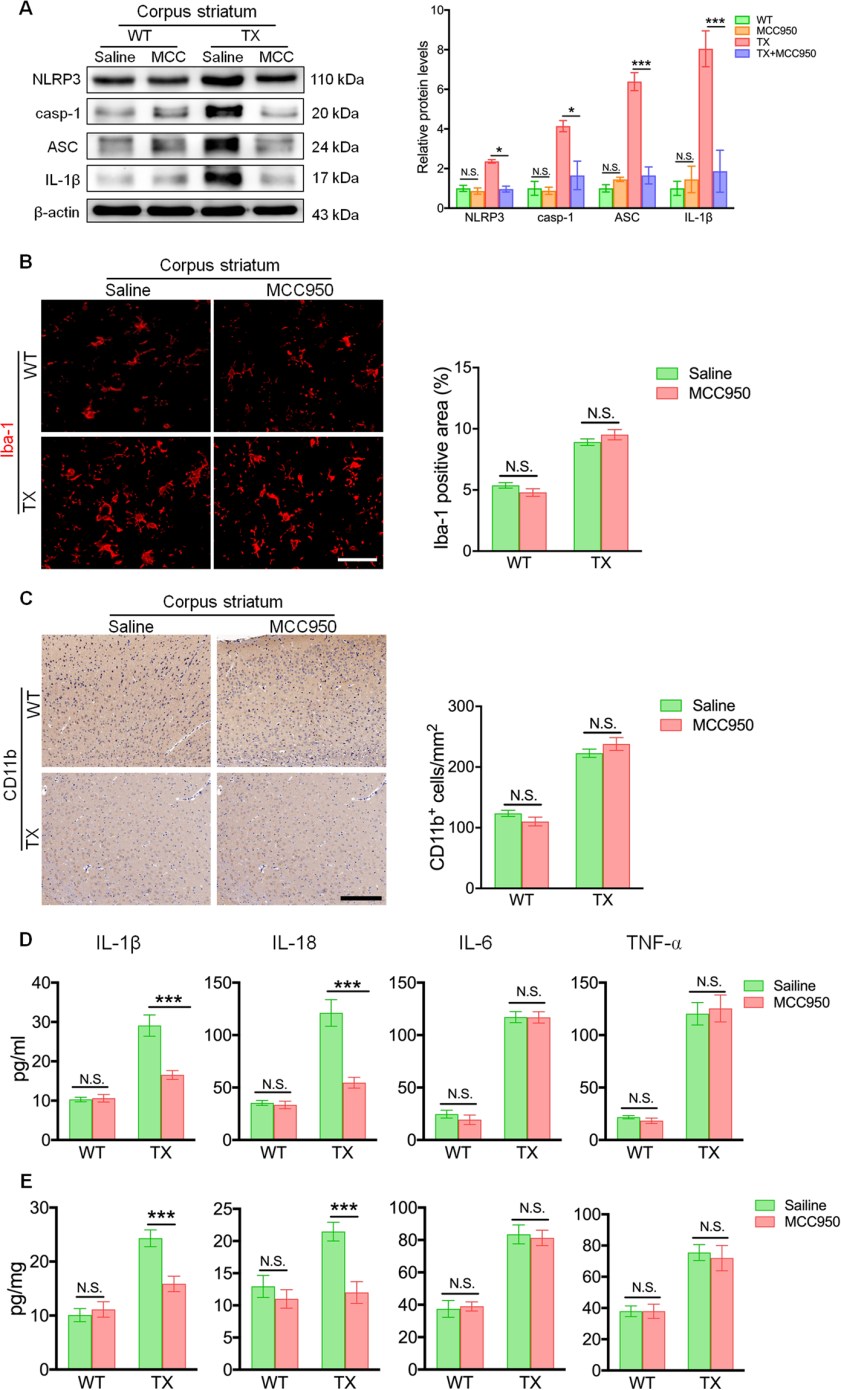
MCC950 inhibits NLRP3 inflammasome activation in the Wilson’s disease (WD) animal model. **a** Levels of NLRP3, cleaved caspase-1 (casp-1), ASC, and interleukin (IL)-1β in the corpus striatum of MCC950 or saline-treated toxic milk (TX) mice and wild type (WT) mice were quantified using western blotting (*n* = 3). **b** Immunofluorescence staining and quantification of Iba-1positive cells in the corpus striatum of MCC950 or saline-treated TX mice and WT mice. Scale bar, 50 µm. **c** Immunohistochemical staining and quantification of CD11b-positive cells in the corpus striatum of TX mice and WT mice treated with MCC950 or saline. Scale bars, 200 µm. **d** Serum IL-1β, IL-18, IL-6, and tumor necrosis factor (TNF)-α levels in TX mice and WT mice treated with MCC950 or saline (pg/ml) (*n* = 6). **e** IL-1β, IL-18, IL-6, and TNF-α levels in the brain of TX mice and WT mice treated with MCC950 or saline (pg/mg) (*n* = 6). Data are presented as mean ± SEM; two-way ANOVA, ^*^P < 0.05, ^***^P < 0.001 versus the corresponding saline-treated group.

**Fig. 8:**
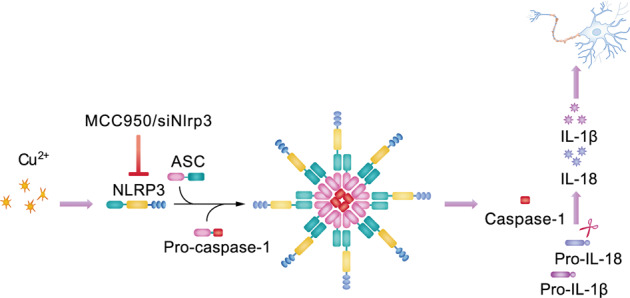
Inhibiting NLRP3 inflammasome activation prevents copper-induced neuropathology in Wilson’s disease (WD) animal model. NLRP3 inflammasome activation is actively involved in the progression of WD and its inhibition protects against Cu^2+^-induced neuroinflammation and prevents neuronal injury.

Next, we evaluated whether MCC950 had any impact on inflammasome activation. Mice treated with MCC950 showed markedly decreased levels of NLRP3, cleaved caspase-1, and IL-1β in the corpus striatum (Fig. 7a) and other areas, including the hippocampus, cortex, and cerebellum (Fig. S9), as compared to those in saline-treated animals. MCC950 decreased the formation and release of ASC in TX mice compared to that in saline-treated animals (Fig. 7a and Fig. S9). The numbers of activated microglia were significantly increased in the brains of TX mice, whereas MCC950 treatment did not affect the number of activated microglia in different brain regions of TX mice (Fig. 7b, c, and Figs. S10, S11). In addition, the levels of IL-1β and IL-18 in the serum and brain were reduced in MCC950-treated TX mice (Fig. 7d, e). However, the MCC950 administration did not affect the levels of IL-6 and TNF-α in the serum and brain of TX mice (Fig. 7d, e). Altogether, these observations indicated that MCC950 suppresses neuroinflammation and protects against neurodegeneration in vivo by inhibiting NLRP3 inflammasome activation in TX mice (Fig. 8).

## Discussion

WD is caused by abnormal copper metabolism in the liver due to a mutation in the gene encoding the copper transporting protein ATP7B. Current treatments for WD, including copper chelating agents, are designed to remove excess copper from tissues and organs^27^. Although these treatments have been used for decades, they are not entirely effective in many patients^28^. Therefore, the development of auxiliary therapies is required to improve the efficacy of copper chelation. Furthermore, chronic neuroinflammation reportedly plays a pivotal role in the pathogenesis of WD. In this study, we demonstrated that the NLRP3 inflammasome is actively involved in WD pathophysiology and progression. In a murine model of WD, NLRP3 silencing or pharmacologic inhibition of NLRP3 inflammasome activation attenuated the pathological progression of copper-induced injury and prevented neurodegeneration.

Acute inflammatory cytokines, such as TNF-α, and chronic inflammatory signals, such as inflammasome activation, have been implicated in neurological inflammatory conditions. Expectedly, major NLRP3 inflammasome markers IL-1β and IL-18 were elevated in the sera of WD patients, suggesting an essential role for inflammasome dysregulation in the development of neuroinflammation in WD. Various types of inflammasomes, such as NLRC4, NLRP3, NLRP2, NLRP1, and AIM2, were activated in different regions of the brain in TX mice. Among these, activation of the NLRP3 inflammasome resulted in the most substantial changes. In addition, activation of the canonical NLRP3 inflammasome is more sensitive to accumulated copper in the brain, as compared to other inflammasomes. Further, a copper chelator inhibited the NLRP3 and not the AIM2, NLRC4, and NLRP1 inflammasomes or NF-κB-dependent priming^25^. Therefore, we speculate that Cu^2+^ specifically triggers the intracellular NLRP3 sensor to activate the inflammasome; however, the detailed mechanism remains to be further investigated.

It is widely accepted that successful NLRP3 inflammasome activation requires co-operation between NF-κB-activating stimuli, such as toll-like receptor ligands, to transcriptionally enhance the expression of pro-IL-1β^29,30^. Moreover, the activating signal is provided by various endogenous stimuli, such as PAMP or Cu^2+^-conjugated misfolded proteins, which activate NLRP3^31–33^. Unlike several other NLRP3 inhibitors, MCC950 inhibits NLRP3-induced ASC oligomerization and prevents caspase-1 cleavage into its active form, with specific selectivity^34,35^. Notably, the potential therapeutic value of MCC950 has been validated in vivo in numerous disease models^36–38^. MCC950 was found to effectively block NLRP3 inflammasome activation and protect against cognitive decline, motor deficits, and neurodegeneration in TX mice. Interestingly, MCC950 did not inhibit the expression of classically secreted proinflammatory cytokines, such as TNF-α and IL-6; however, it only reduced unconventionally released caspase-1-dependent cytokines such as IL-1β and IL-18. The results demonstrate that MCC950 inhibits the assembly of the inflammasome, and this is indicated by its ability to prevent the expression of caspase 1 and ASC in the WD animal model.

Microglia, resident immune cells in the brain, could be activate by copper to induce the inflammatory response. Microglial activation is an important characteristic of the brain in WD and in animal models of WD, and the over-expression of Iba-1 indicates this change^4,39^. Reportedly, NLRP3 inflammasome activation plays a critical role in the initiation of microglia-mediated neuroinflammation and neuronal degeneration. Intriguingly, our analysis of microglia numbers inhibited by the activation of NLRP3 inflammasome in WD mice showed no significant differences. It is also possible that, in addition to NLRP3, other inflammasomes participate in copper-induced neuropathology. Therefore, a combination of an inflammasome activation inhibitor, such as MCC950, and a microglial activation inhibitor, such as minocycline, may be more effective for treating neurodegeneration in TX mice.

## Conclusions

Altogether, our study provides several lines of evidence to show that NLRP3 inflammasome activation is actively involved in the progression of WD and that its inhibition protects against Cu^2+^-induced neuroinflammation and prevents neuronal injury. Therefore, pharmacologically targeting NLRP3 inflammasome activation could be a potential therapeutic strategy for treating Cu^2+^-induced neuropathology in WD.

## Material and methods

### Patients and controls

Participants were recruited from the Institute of Neurology, Affiliated Hospital, Anhui University of Chinese Medicine. All patients fulfilled the criteria for the clinical diagnosis of WD. Participants who had disease complications that were associated with inflammasome activation (such as depression or hypercholesterolemia) were excluded from the study. Age-matched healthy individuals (with normal serum ceruloplasmin and copper levels) were recruited as controls. Blood samples of participants were collected and centrifuged at 2500 × *g* for 10 min, and 500 µL of the supernatant was immediately collected and stored at −80 °C for further analysis. All experiments were approved by and performed in accordance with the guidelines of the institutional review board of the Affiliated Hospital of the Neurology Institute of Anhui University of Chinese Medicine. Written informed consent was obtained from all participants recruited in the study.

### Animals

Male and female C3HeB/FeJ (WT) and C3HeB/FeJ-Atp7btx-J/J (TX) mice^40^ were purchased from the Jackson Laboratory (Bar Harbor, ME, USA). All mice were housed at the Hefei Institutes of Physical Science, Chinese Academy of Sciences facility under standard laboratory conditions (12-h light/dark cycle, 20–22 °C, 50–60% humidity), with ad libitum access to water and food throughout the study duration. All in vivo experiments were performed with 10- to 12-week-old animals; weighing 25–30 g. Ethical approval for the animal experiments was obtained from the Hefei Institutes of Physical Science, Chinese Academy of Sciences Animal Ethics Committees. Animals were handled in accordance with the National Institutes of Health Guide for the Care and Use of Laboratory Animals.

### Generation of *Nlrp3* knockdown mice

For *Nlrp3* knockdown, either lentivirus-enveloped Nlrp3 siRNA (siNlrp3) (Genepharma, Shanghai, China) or lentivirus-enveloped negative control siRNA (NC) were injected through the tail vein at a dosage of 20 μL/mouse (10^9^ TU/mL). For blockade of NLRP3 inflammasome activation, 20 mg/kg of MCC950 (Selleck, Houston, TX, USA) was administered daily via oral gavage for 30 days. Control animals were given an equal volume of phosphate buffered saline (PBS).

### Cell culture and cell viability assay

Primary microglia and neuron were prepared as previously described^41,42^. To activate the NLRP3 inflammasome, primary microglia were incubated with 200 ng/mL of ultrapure LPS (Sigma, St. Louis, USA) for 3 h and NLRP3 inflammasome activation was stimulated with 5 mM adenosine 5′-triphosphate (ATP; Sigma) or 10 µM CuCl_2_ (Sigma) for 1 h (unless indicated otherwise). The microglia-conditioned media were collected, centrifuged at 900 × *g* for 10 min, and transferred into primary neuron culture. For the inhibition study, MCC950 (100 nM) was added after the priming step. The survival of neurons was measured by Cell Counting Kit-8 (CCK-8; Dojindo, Kumamoto, Japan) according to the manufacturer’s instruction.

### ELISA for inflammatory cytokines

Mice were euthanized with an overdose of ketamine hydrochloride and transcardially perfused with 20 mL cold PBS as described previously prior to the removal of brain tissue^43^. Thereafter, brain tissues were then homogenized using an automatic grinder (Servicebio, Wuhan, China). The concentrations of inflammatory cytokines including IL-1β (Invitrogen, Vienna, Austria), IL-18 (Invitrogen), tumor necrosis factor (TNF-α; Invitrogen), and IL-6 (Invitrogen) were quantified using commercial ELISA kits following the manufacturer’s instructions. The absorbance was read at 450 nm using a microplate reader (BioTek, VT, USA).

### Immunohistochemical staining

Immunohistochemical staining was performed as described previously^44^. Briefly, mouse brains were transcardially perfused and subsequently fixed with 4% paraformaldehyde overnight. The brain tissues were sectioned through the region of interest at a thickness of 20–30 μm, and endogenous peroxidase activity was blocked with 3% H_2_O_2_. Thereafter, sections were incubated with anti–CD11b antibody (1:4000, Abcam, Cambridge, UK) at 4 °C overnight, rinsed three times with PBS, and incubated with the secondary antibody at 25 ± 1 °C for 1 h. For fluorescent staining, sections were blocked with 3% bovine serum albumin (BSA) for 30 min, and subsequently incubated with anti-Iba-1 antibody (1:100; Abcam), and anti-NeuN antibody (1:100; Abcam) at 4 °C overnight. Thereafter, sections were rinsed thrice in PBS and incubated with fluorescent-labeled secondary antibodies at 25 ± 1 °C for 1 h in the dark. Samples were visualized using Olympus BX53 microscope (Olympus, Tokyo, Japan), and the images analyzed using Image J (NIH, Bethesda, MD, USA).

### TUNEL staining

Apoptotic neuronal cells were quantified using Fluorescein Tunel Cell Apoptosis Detection Kit (Servicebio) following the manufacturer’s instructions and as previously described^45^. The samples were visualized using BX53 microscope (Olympus) by an experienced pathologist blinded to the experimental design.

### Statistical analyses

GraphPad Prism 8 (GraphPad, La Jolla, CA, USA) was used to perform all statistical analyses. All data were expressed as mean ± standard error of the mean. Student’s *t* test was used for single variant analyses. The differences between means among multiple groups were compared using one-way or two-way analysis of variance (ANOVA); a *P*-value < 0.05 was considered significant.

### Other methods

Detailed description of other methods used in this study is provided in Supplementary Materials and Methods.

## Supplementary information

Supplementary figure legends

Figure S1

Figure S2

Figure S3

Figure S4

Figure S5

Figure S6

Figure S7

Figure S8

Figure S9

Figure S10

Figure S11

Supplementary Material and Methods
