# Supplementary material for: Inhibiting NLRP3 inflammasome activation prevents copper-induced neuropathology in a murine model of Wilson’s disease
Source: Cell Death Dis. 2021 Jan 18;12(1):87. doi: 10.1038/s41419-021-03397-1 (PMC7813851; doi:10.1038/s41419-021-03397-1)
Supplement: Supplementary file 1 — Supplementary figure legends [file 41419_2021_3397_MOESM1_ESM.doc]

**Supplementary figure legends**

**Fig. S1 Expression of inflammasome components in the brain of Wilson’s disease (WD) animal model.** Levels of cleaved caspase-1 (casp-1), ASC, and interleukin (IL)-1β in the (a) hippocampus, (b) cortex, and (c) cerebellum of toxic milk (TX) mice and wild type (WT) mice were quantified using western blotting (n = 6 each) Data are presented as mean ± SEM; Student’s *t* test, *^*^P* < 0.05, *^**^P* < 0.01, *^***^P* < 0.001 versus the corresponding WT group.

**Fig. S2 Activation of NLRP3 inflammasome in the brain of Wilson’s disease (WD) animal model**. (a) Levels of NLRP1, NLRP2, NLRP3, NLRC4, and AIM2 in the (a) hippocampus, (b) cortex, and (c) cerebellum of toxic milk (TX) mice and wild type (WT) control mice were quantified using western blotting (n = 6 each). Data are presented as mean ± SEM; Student’s *t* test, *^**^P*<0.01, *^***^P*<0.001 versus the corresponding WT group.

**Fig. S3 Gene expression of inflammasome components in the brain of Wilson’s disease (WD) animal model.** Expression of *Nlrp3*, *casp-1*, and *ASC* in the (a) corpus striatum, (b) hippocampus, (c) cortex, and (d) cerebellum of toxic milk (TX) mice and wild type (WT) mice (n = 6 each). Data are presented as mean ± SEM; Student’s *t* test, *^*^P* < 0.05, *^**^P* < 0.01, *^***^P* < 0.001 versus the corresponding WT group.

**Fig. S4 Activation of NLRP3 inflammasome in the brain of *Nlrp3*-silenced (siNlrp3-treated) Wilson’s disease (WD) animal model.** Levels of NLRP3, cleaved caspase-1 (casp-1), ASC, and interleukin (IL-1β) in the (a) hippocampus, (b) cortex, and (c) cerebellum of negative control siRNA (NC)- or siNlrp3-injected toxic milk (TX) mice and wild type (WT) mice were quantified using western blotting (n = 3 each). Data are presented as mean ± SEM; two-way ANOVA, *^*^P* < 0.05, *^**^P* < 0.01, *^***^P* < 0.001 versus the corresponding WT or NC group.

**Fig. S5 Microglia numbers in the brain of Wilson’s disease (WD) animal model following siRNA administration.** Immunofluorescence staining and quantification of Iba-1-positive cells in the (a) hippocampus, (b) cortex, and (c) cerebellum of negative control siRNA (NC)- or siNlrp3-injected toxic milk (TX) mice and wild type (WT) mice. Scale bars, 50 µm. Data are presented as mean ± SEM; two-way ANOVA, *^*^P* < 0.05, *^**^P* < 0.01, *^***^P* < 0.001 versus the corresponding NC group.

**Fig. S6 Activation microglia numbers in the brain of Wilson’s disease (WD) animal model following siRNA administration.** Immunohistochemical staining and quantification of CD11b-positive cells in the (a) hippocampus, (b) cortex, and (c) cerebellum of negative control siRNA (NC)- or siNlrp3-injected toxic milk (TX) mice and wild type (WT) mice. Scale bars, 200 µm. Data are presented as mean ± SEM; two-way ANOVA, *^*^P* < 0.05, *^**^P* < 0.01, *^***^P* < 0.001 versus the corresponding NC group.

**Fig. S7 MCC950 prevents neuronal degradation in the brain of Wilson’s disease (WD) animal model**. Immunofluorescence and quantification of Fluoro-Jade B (FJB) staining (green) in the (a) hippocampus, (b) cortex, and (c) cerebellum of MCC950- or saline-treated toxic milk (TX) mice and wild type (WT) mice. Scale bars, 50 µm. Data are presented as the mean ± SEM; two-way ANOVA, *^*^P* < 0.05, *^**^P* < 0.01 versus the corresponding saline-treated group.

**Fig. S8 MCC950 treatment reduces neuron loss in the brain of Wilson’s disease (WD) animal model.** Immunofluorescence staining and quantification of NeuN in the (a) hippocampus, (b) cortex, and (c) cerebellum of toxic milk (TX) mice and wild type (WT) mice treated with MCC950 or saline. Scale bars, 50 µm. Data are presented as mean ± SEM; two-way ANOVA, *^**^P* < 0.01, *^***^P* < 0.001 versus the corresponding saline-treated group.

**Fig. S9 MCC950 inhibits NLRP3 inflammasome activation in the brain of Wilson’s disease (WD) animal model.** Levels of NLRP3, cleaved caspase-1 (casp-1), ASC, and interleukin (IL-1β) in the (a) hippocampus, (b) cortex, and (c) cerebellum of MCC950- or saline-treated toxic milk (TX) mice and wild type (WT) mice were quantified using western blotting (n = 3 each). Data are presented as mean ± SEM; two-way ANOVA, *^*^P* < 0.05, *^**^P* < 0.01, *^***^P* < 0.001 versus the corresponding saline-treated group.

**Fig. S10 MCC950 has no effects on microglia activation in the Wilson’s disease (WD) animal model.** Immunofluorescence and quantification of Iba-1-positive cells in the (a) hippocampus, (b) cortex, and (c) cerebellum of toxic milk (TX) mice and wild type (WT) mice treated with MCC950 or saline. Scale bars, 50 µm. Data are presented as mean ± SEM; two-way ANOVA, *^*^P* < 0.05, *^**^P* < 0.01, *^***^P* < 0.001 versus the corresponding saline-treated group.

**Fig. S11 Activation microglia numbers in the brain of Wilson’s disease (WD) animal model following MCC950 administration.** Immunohistochemical staining and quantification of CD11b-positive cells in the (a) hippocampus, (b) cortex, and (c) cerebellum of toxic milk (TX) mice and wild type (WT) mice treated with MCC950 or saline. Scale bars, 200 µm. Data are presented as mean ± SEM; two-way ANOVA, *^*^P* < 0.05, *^**^P* < 0.01, *^***^P* < 0.001 versus the corresponding saline-treated group.
