# Supplementary figures and images for: Inhibiting NLRP3 inflammasome activation prevents copper-induced neuropathology in a murine model of Wilson’s disease
Source: Cell Death Dis. 2021 Jan 18;12(1):87. doi: 10.1038/s41419-021-03397-1 (PMC7813851; doi:10.1038/s41419-021-03397-1)

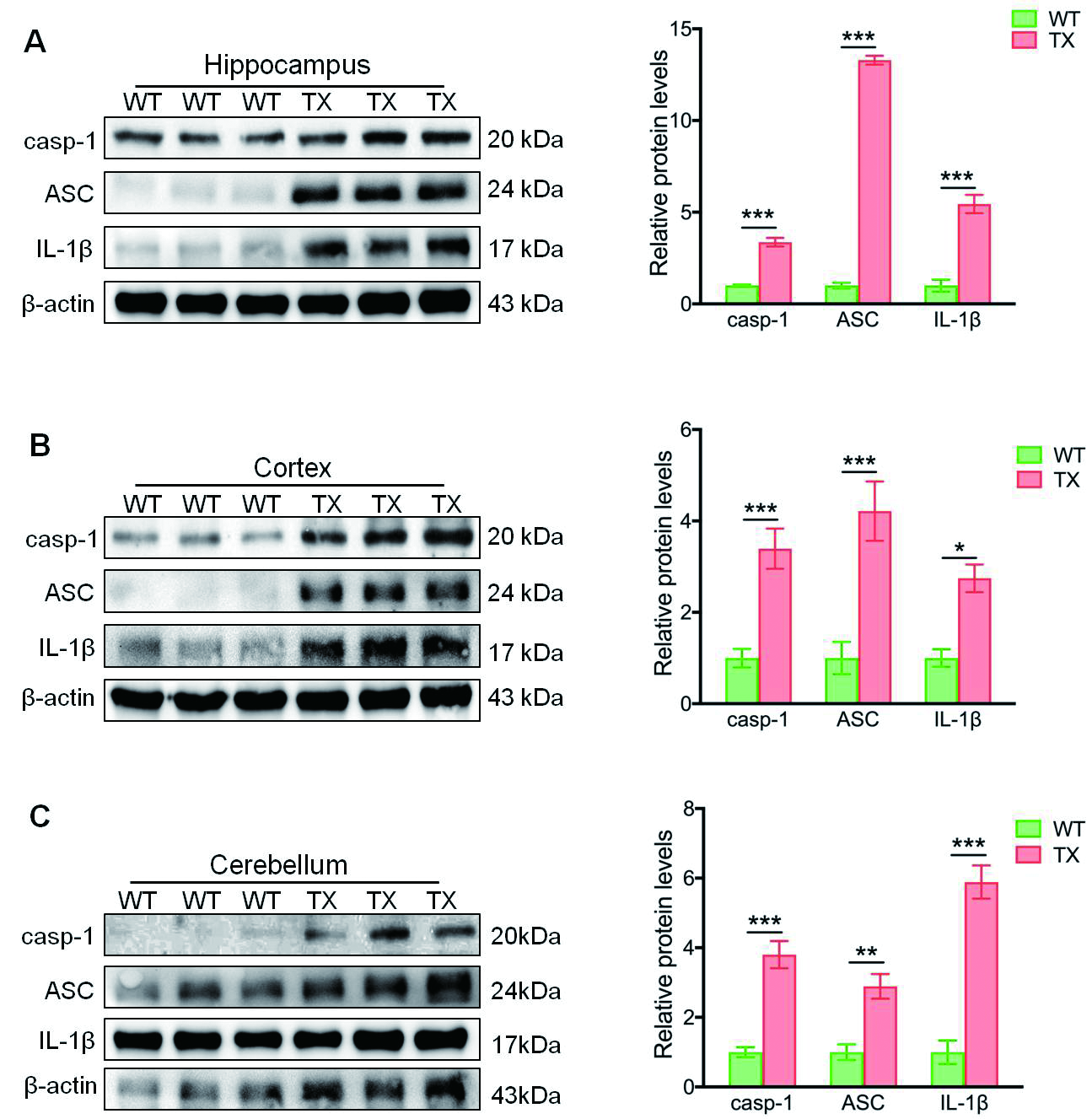

Supplement: Supplementary file 2 — Figure S1 [file 41419_2021_3397_MOESM2_ESM.tif]

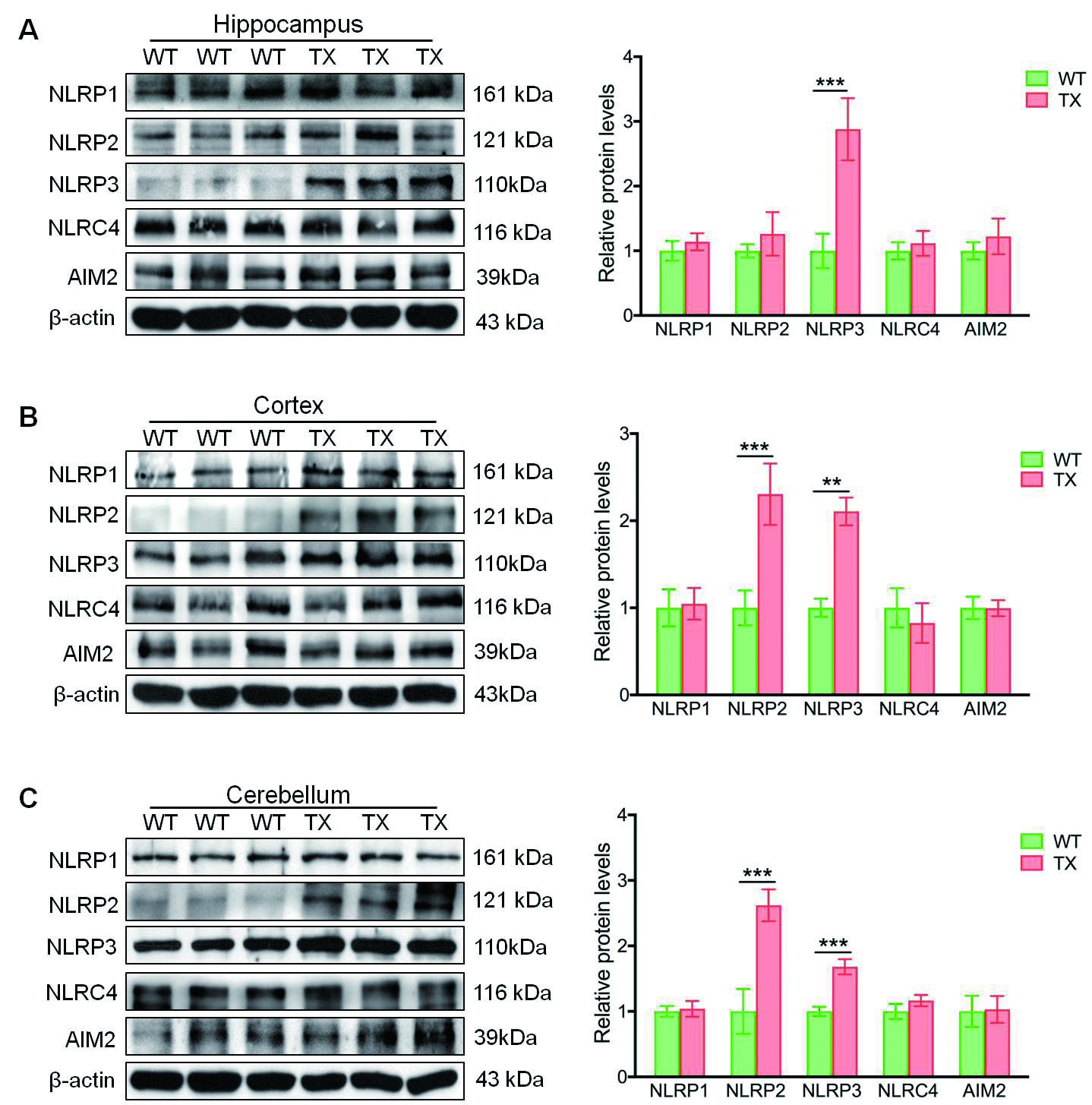

Supplement: Supplementary file 3 — Figure S2 [file 41419_2021_3397_MOESM3_ESM.tif]

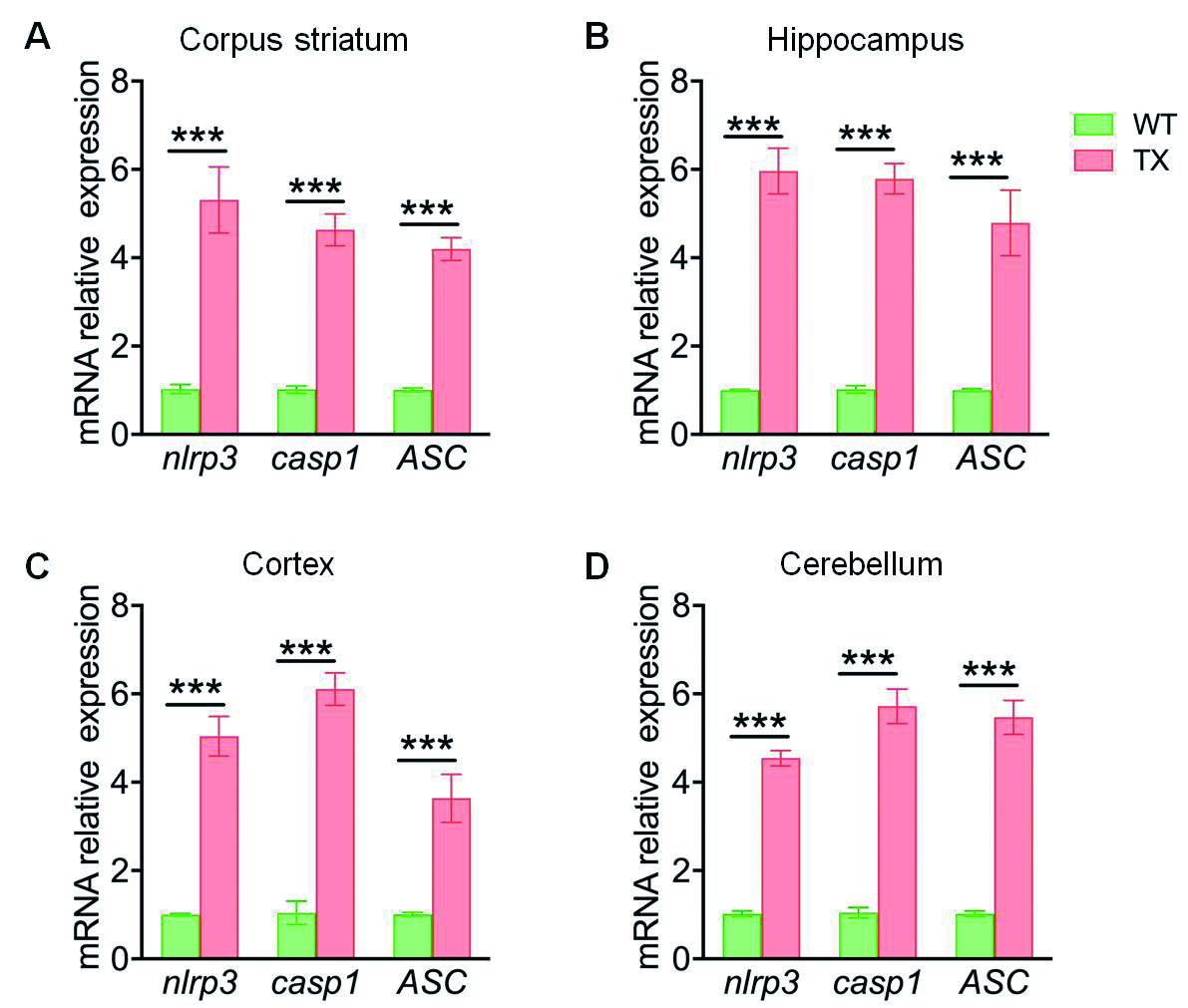

Supplement: Supplementary file 4 — Figure S3 [file 41419_2021_3397_MOESM4_ESM.tif]

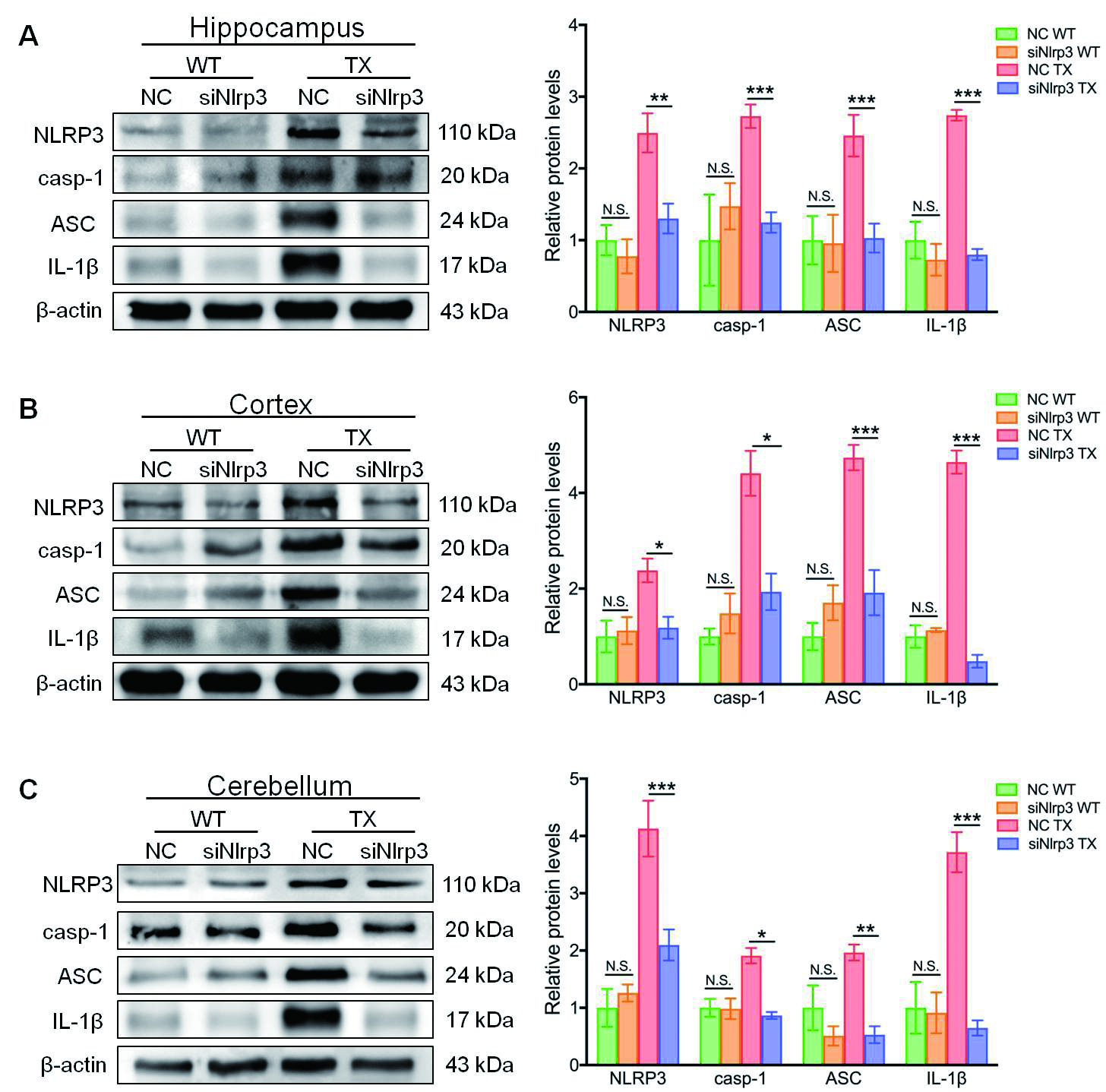

Supplement: Supplementary file 5 — Figure S4 [file 41419_2021_3397_MOESM5_ESM.tif]

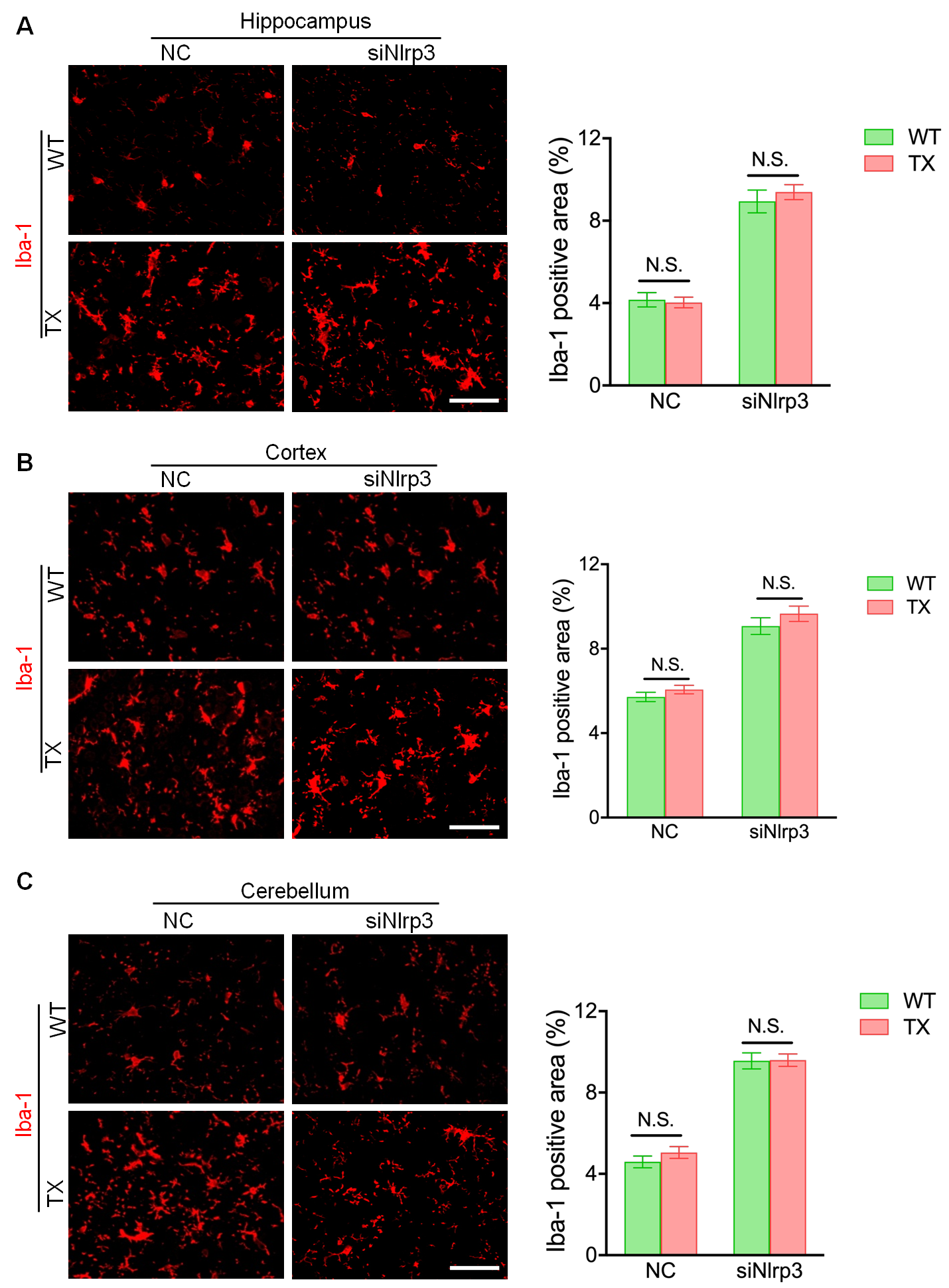

Supplement: Supplementary file 6 — Figure S5 [file 41419_2021_3397_MOESM6_ESM.tif]

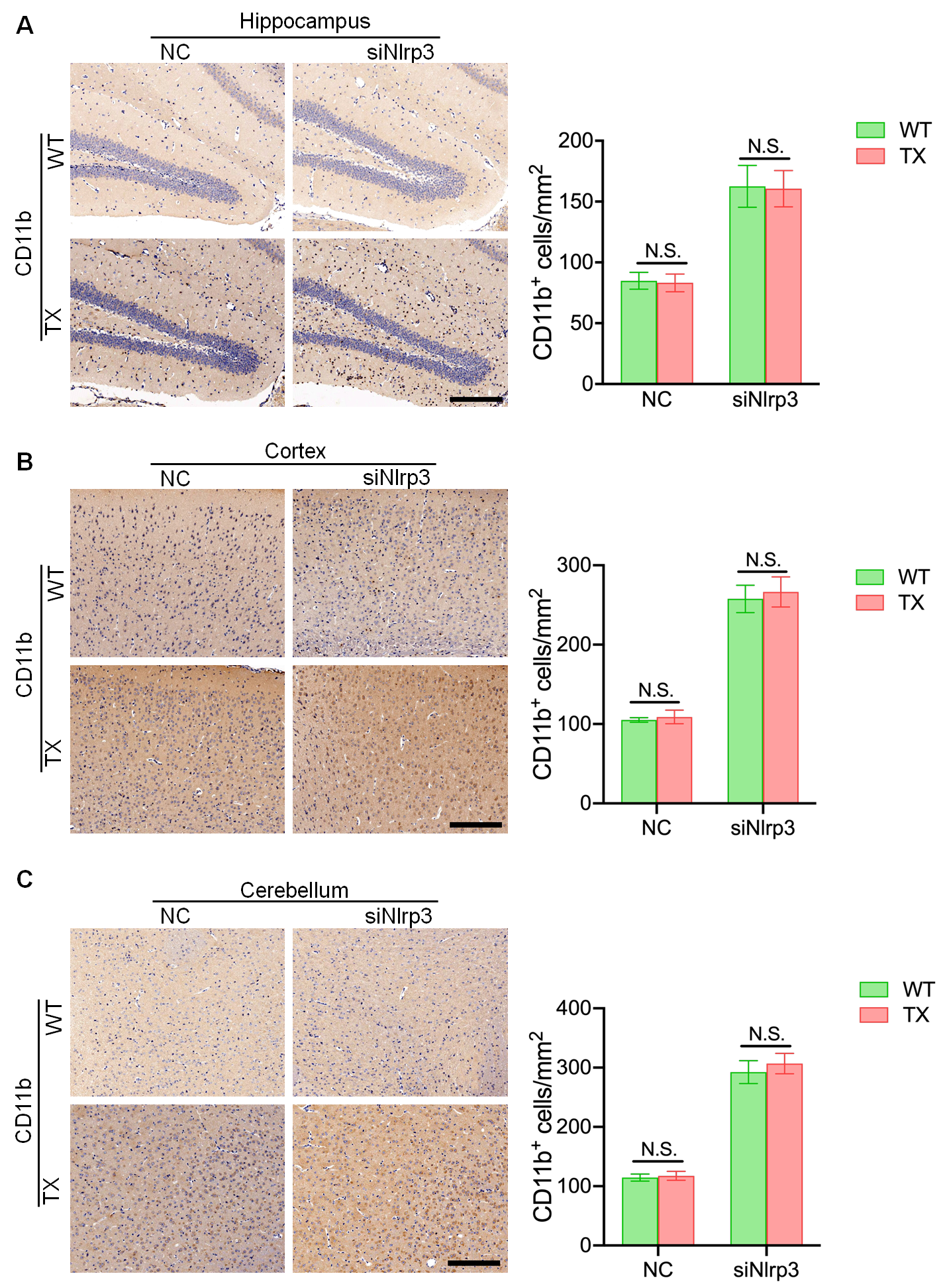

Supplement: Supplementary file 7 — Figure S6 [file 41419_2021_3397_MOESM7_ESM.tif]

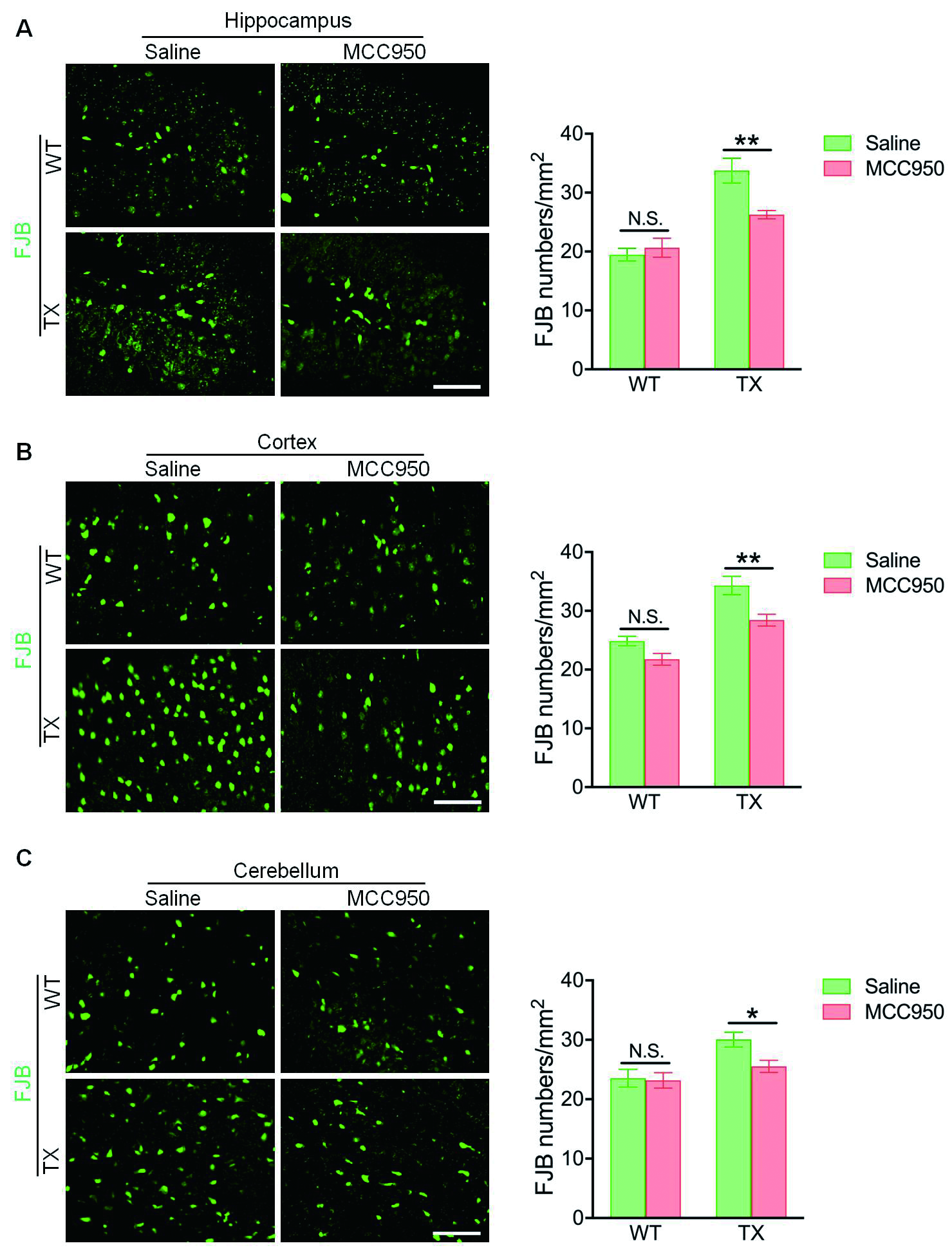

Supplement: Supplementary file 8 — Figure S7 [file 41419_2021_3397_MOESM8_ESM.tif]

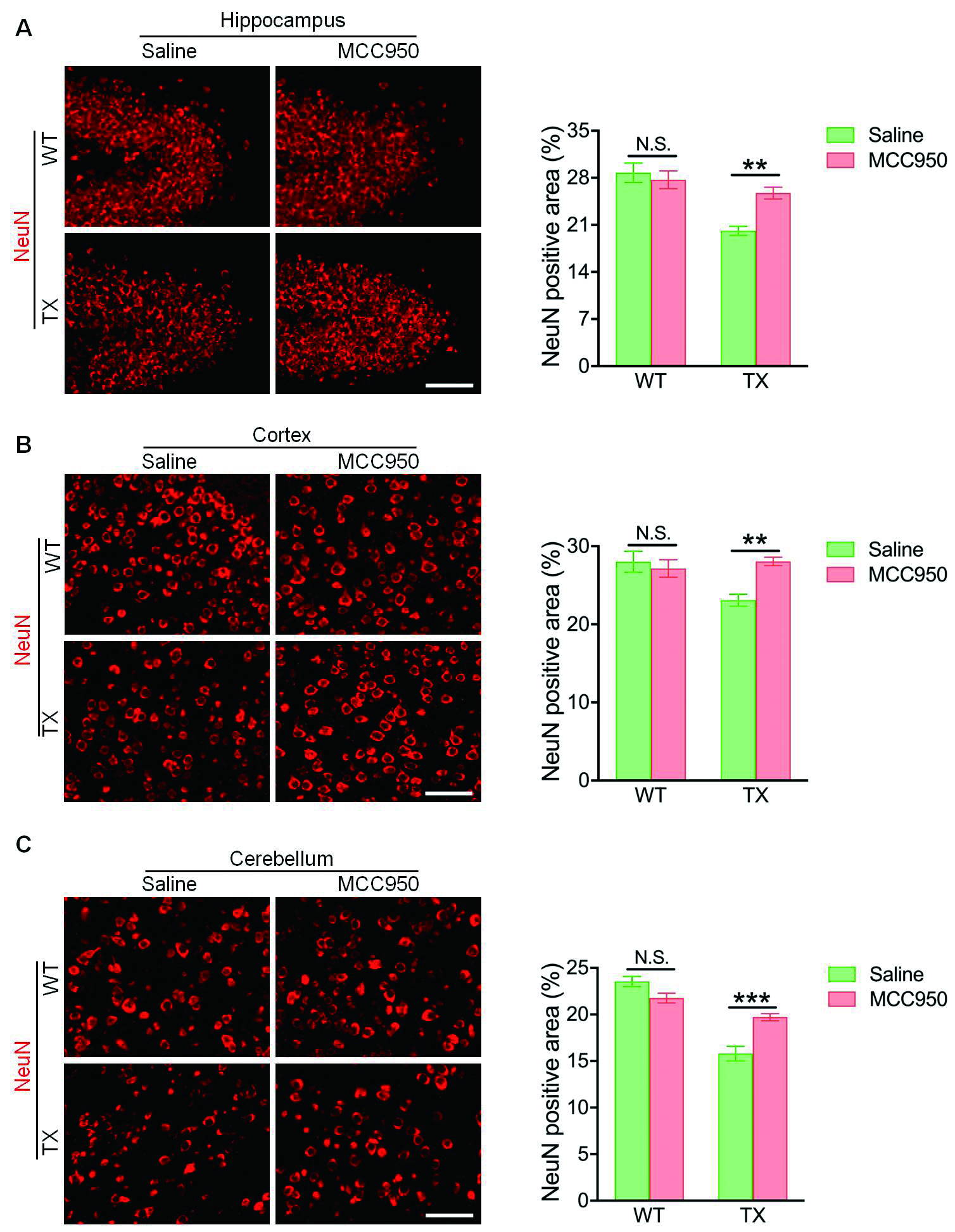

Supplement: Supplementary file 9 — Figure S8 [file 41419_2021_3397_MOESM9_ESM.tif]

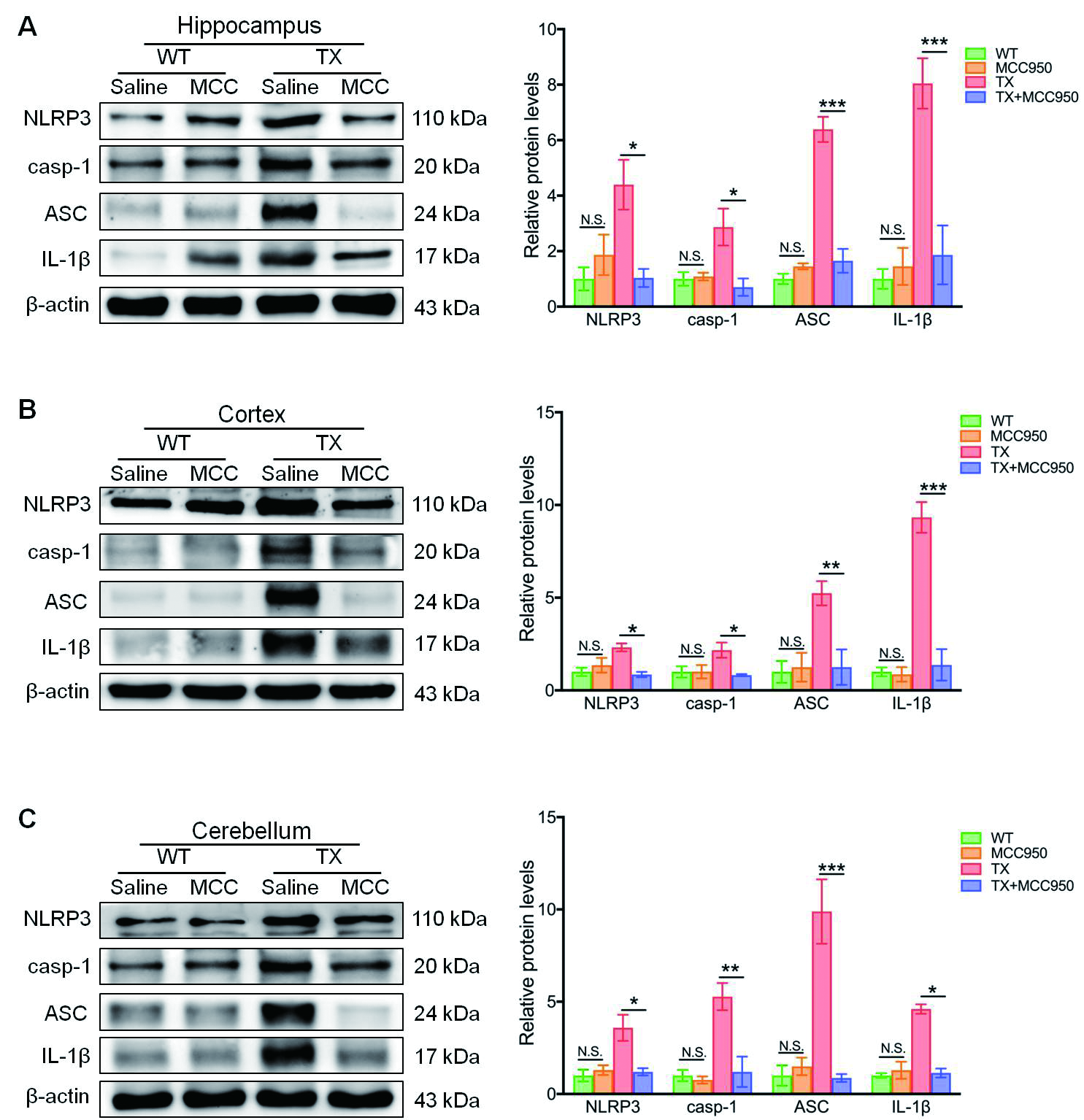

Supplement: Supplementary file 10 — Figure S9 [file 41419_2021_3397_MOESM10_ESM.tif]

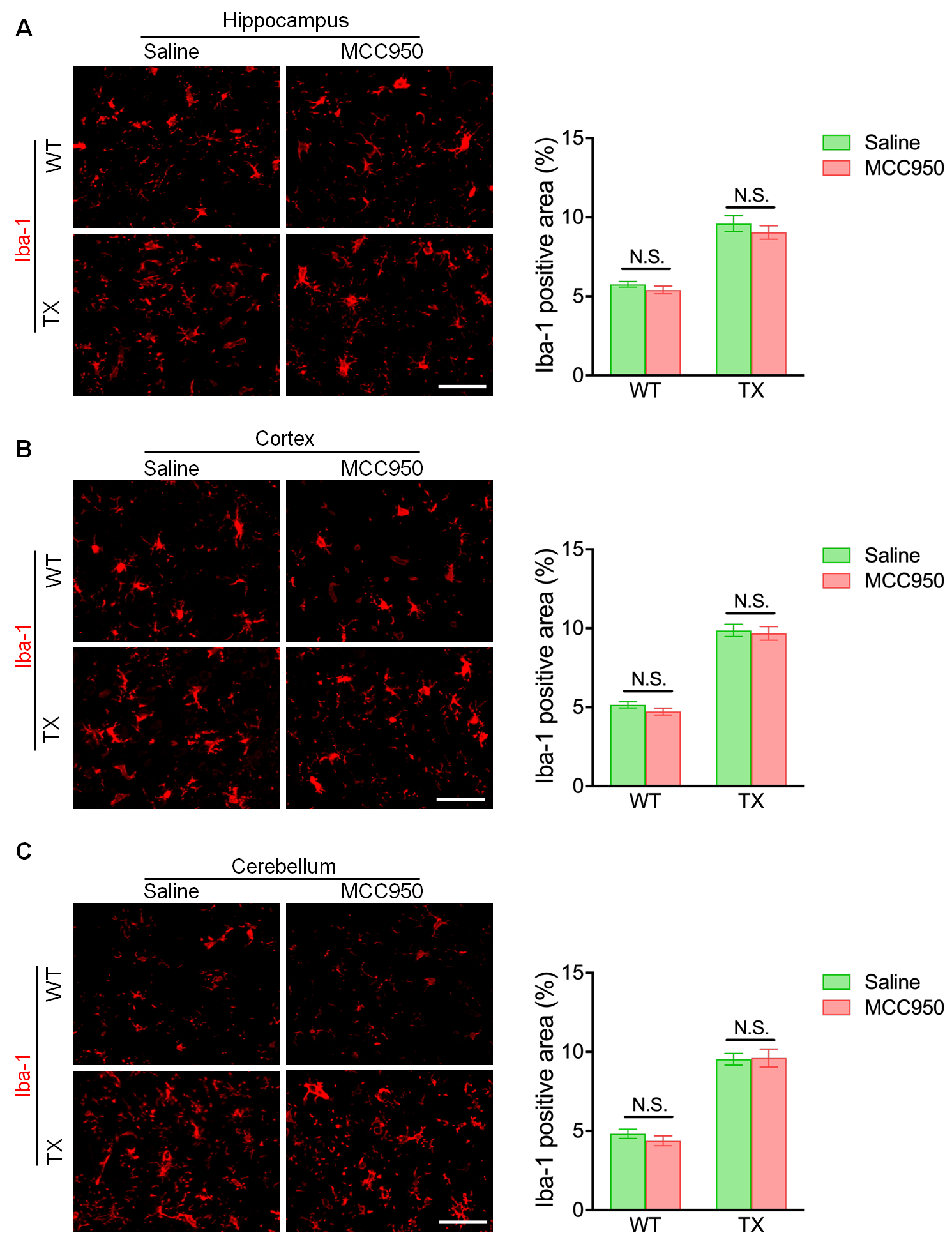

Supplement: Supplementary file 11 — Figure S10 [file 41419_2021_3397_MOESM11_ESM.tif]

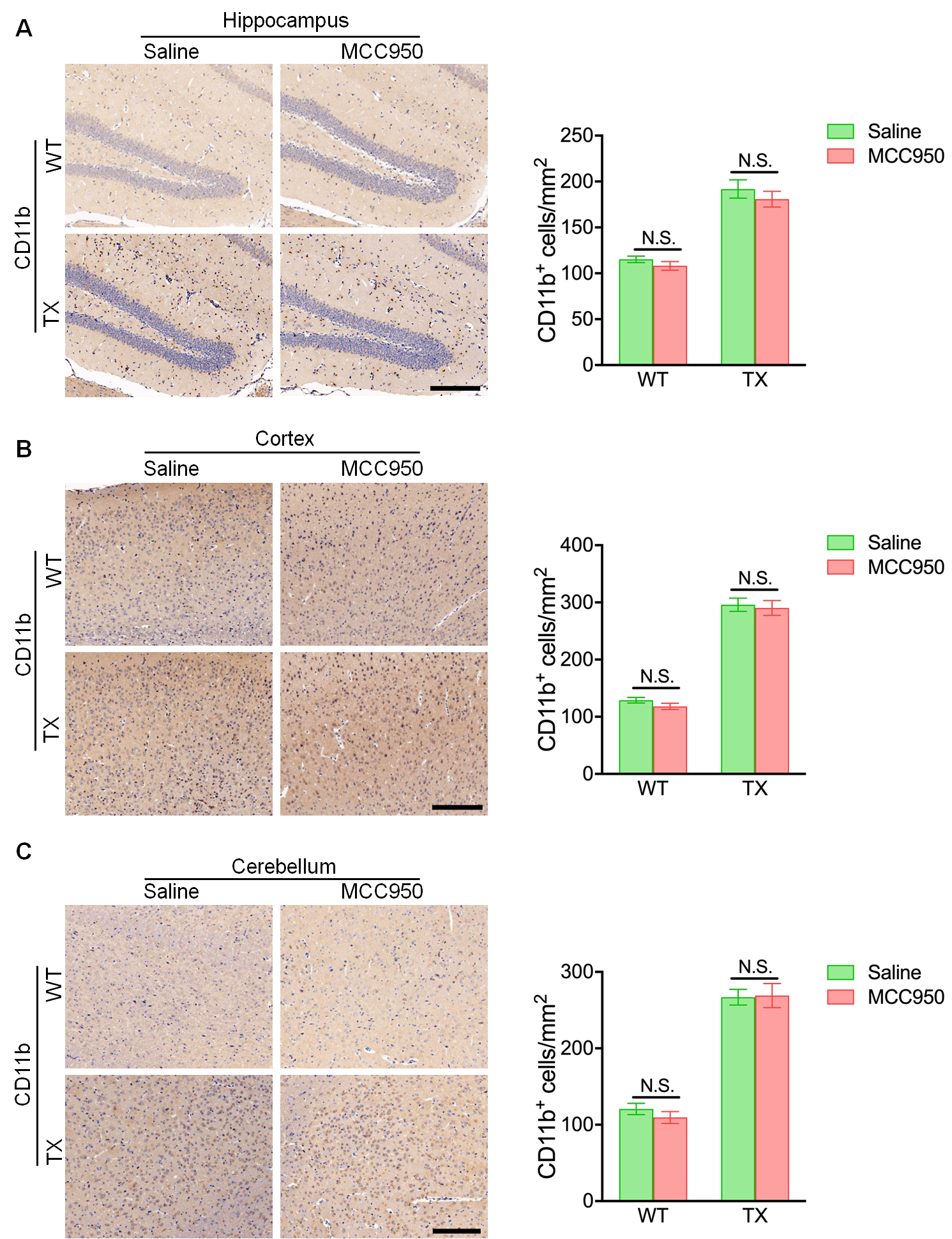

Supplement: Supplementary file 12 — Figure S11 [file 41419_2021_3397_MOESM12_ESM.tif]
