# Supplementary material for: Inhibiting NLRP3 inflammasome activation prevents copper-induced neuropathology in a murine model of Wilson’s disease
Source: Cell Death Dis. 2021 Jan 18;12(1):87. doi: 10.1038/s41419-021-03397-1 (PMC7813851; doi:10.1038/s41419-021-03397-1)
Supplement: Supplementary file 13 — Supplementary Material and Methods [file 41419_2021_3397_MOESM13_ESM.docx]

**Supplementary Material and Methods**

**Fluoro-Jade B staining (FJB)**

The brain slides were incubated with FJB working solution (0.1% acetic acid) at 4 °C overnight, rinsed with distilled water, and dried in an oven at 50–60 °C for 15 min. Finally, the sections were visualized, and the number of FJB-positive cells was counted under BX53 microscope (Olympus).

**RNA extraction and quantitative RT-PCR**

Total RNA was isolated using the RNAprep Pure Tissue kit (TIANGEN, Beijing, China) according to manufacturer’s instructions. cDNA was synthesized using the High Capacity cDNA RT kit (QIAGEN, Hilden, Germany). The following primer sequences were used: NLRP3, NM_001359638.1; caspase 1, NM_009807.2; ASC, NM_023258.4. *β-actin* was used as an internal control. Quantitative real-time PCR was performed using SYBR premix (QIAGEN) on a StepOne Plus real-time PCR system (ABI, Darmstadt, Germany).

**Western blot analysis**

Cultured microglia or brain tissues were lysed using RIPA buffer (Solarbio, Beijing, China). Approximately 20–30 µg protein was separated using a 10%–15% SDS-polyacrylamide gel and transferred onto a 0.2-µm NC-membrane (GE Healthcare Life Sciences, Pittsburgh, PA, USA). The membranes were blocked using a blocking buffer (Beyotime, Shanghai, China) and incubated with primary antibodies at 4 °C overnight. The following primary antibodies were used: anti-NLRP3 (1:1000; Abcam), anti-NLRP1 (1:500; Santa Cruz), anti-NLRP2 (1:500; Santa Cruz), anti-NLRC4 (1:1000; Abcam), anti-AIM (1:500; Santa Cruz), anti-ASC (1:500; Santa Cruz), anti-caspase 1 (1:1000; Abcam), and anti-IL-1β (1:1000; CST). The membranes were then washed 4–5 times and incubated with anti-rabbit, anti-goat, or anti-mouse horseradish peroxidase (HRP)-conjugated secondary antibodies (1:10000; Abcam) in 2% BSA/TBS-T at 25 ± 1 °C for 1–2 h. The signal was detected with ECL (Thermo Scientific), and β-actin was used as a loading control.

**Behavioral analysis**

All behavioral examinations were conducted during the light phase of the light/dark circle. Mice were acclimatized to the laboratory environment for at least 30 min before each test. Three different behavior examinations were conducted with the following sequence OFT-RT-BM, spaced by 24 h.

**Open-field test (OFT)**

Briefly, mice were placed in the center of the novel open-field arena (50 cm long, 40 cm wide, and 30 cm tall) and examined using an automatic video tracking system (XinRuan, Shanghai, China) for 5 min. Parameters, including rearing numbers and travel distance, were analyzed to reveal the travel path followed by the mice.

**Rotarod test**

Mice were first trained on the Rotarod apparatus (XinRuan) at an increasing speed starting from 4 rpm and accelerating to 40 rpm for 5 min over 2 consecutive days. Mice received three trials per day during the training and testing, and latency to fall was recorded at a speed of 40 rpm. The average of three latency to fall experiments was used to estimate motor function.

**Barnes maze**

Mice (n = 15 for WT, and n = 15 for TX mice) were subjected to a habitual experiment to evaluate their memory. On the first day, each mouse was guided to the shelter after placing it on the platform. The mice underwent a space acquisition phase for 4 consecutive days, during which they were allowed to freely explore the test room for 3 min each day. Mice that failed to reach the shelter during the 3 min exploration were manually guided. At day 5, a probe test was performed, where the mice were able to freely explore the maze while all openings, including the one leading to the shelter, were closed. The platform and escape box were cleaned using 70% ethanol after each experiment to eliminate odor. The Noldus Maze Video Tracking System (Noldus, Amsterdam, Netherlands) was used to measure the time each mouse took to reach the escape box.
